# Large language model-based triage to identify antiretroviral therapy adherence barriers and risk levels in patient messages

**DOI:** 10.1093/jamiaopen/ooag169

**Published:** 2026-09-26

**Authors:** Yuanchao Ma, Sofiane Achiche, David Lessard, Kim Engler, Serge Vicente, Gavin Tu, Benoît Lemire, Lina Del Balso, Nathalie Paisible, MARVIN Chatbots Patient Expert Committee, Bertrand Lebouché

**Affiliations:** Institute of Biomedical Engineering, Polytechnique Montreal, Montreal, QC H3T 1J4, Canada; Centre for Outcomes Research & Evaluation, Research Institute of the McGill University Health Centre, Montreal, QC H4A 3J1, Canada; Infectious Diseases and Immunity in Global Health Program, Research Institute of McGill University Health Centre, Montreal, QC H4A 3J1, Canada; Chronic Viral Illness Service, Division of Infectious Disease, Department of Medicine, McGill University Health Centre, Montreal, QC H4A 3J1, Canada; Institute of Biomedical Engineering, Polytechnique Montreal, Montreal, QC H3T 1J4, Canada; Centre for Outcomes Research & Evaluation, Research Institute of the McGill University Health Centre, Montreal, QC H4A 3J1, Canada; Infectious Diseases and Immunity in Global Health Program, Research Institute of McGill University Health Centre, Montreal, QC H4A 3J1, Canada; Chronic Viral Illness Service, Division of Infectious Disease, Department of Medicine, McGill University Health Centre, Montreal, QC H4A 3J1, Canada; Centre for Outcomes Research & Evaluation, Research Institute of the McGill University Health Centre, Montreal, QC H4A 3J1, Canada; Infectious Diseases and Immunity in Global Health Program, Research Institute of McGill University Health Centre, Montreal, QC H4A 3J1, Canada; Département des enseignements généraux, École de technologie supérieure, Université du Québec, Montreal, QC H3C 1K3, Canada; Faculty of Medicine, Université Laval, Quebec, QC G1V 0A6, Canada; Chronic Viral Illness Service, Division of Infectious Disease, Department of Medicine, McGill University Health Centre, Montreal, QC H4A 3J1, Canada; Department of Pharmacy, McGill University Health Centre, Montreal, QC H4A 3J1, Canada; Chronic Viral Illness Service, Division of Infectious Disease, Department of Medicine, McGill University Health Centre, Montreal, QC H4A 3J1, Canada; Chronic Viral Illness Service, Division of Infectious Disease, Department of Medicine, McGill University Health Centre, Montreal, QC H4A 3J1, Canada; Centre for Outcomes Research & Evaluation, Research Institute of the McGill University Health Centre, Montreal, QC H4A 3J1, Canada; Infectious Diseases and Immunity in Global Health Program, Research Institute of McGill University Health Centre, Montreal, QC H4A 3J1, Canada; Chronic Viral Illness Service, Division of Infectious Disease, Department of Medicine, McGill University Health Centre, Montreal, QC H4A 3J1, Canada; Department of Family Medicine, Faculty of Medicine and Health Sciences, McGill University, Montreal, QC H3S 1Z1, Canada

**Keywords:** large language models, medication adherence, natural language processing, HIV, responsible AI, clinical triage

## Abstract

**Objective:**

This study aimed to develop large language models (LLMs) to automatically identify antiretroviral therapy (ART) adherence barriers and stratify nonadherence risk levels from patient-generated messages.

**Materials and Methods:**

With a co-construction committee of people with HIV and providers, 15 480 sentences were annotated for barrier and risk levels. General-domain LLMs (eg, Flan-T5) and clinical foundation models (eg, Clinical-T5) were fine-tuned for multiclass classification and evaluated using Macro-F1. Best-performing models were compared with GPT family and other open-source LLMs. Model fairness, error patterns, and environmental footprints were also assessed.

**Results:**

Flan-T5-xl achieved the best barrier detection (Macro-F1 = 0.83 test/0.71 external), and Flan-T5-large excelled in risk stratification (0.79/0.57). Fine-tuned general-domain LLMs significantly outperformed clinical foundation models (*P* < .001) on the test split. Error analysis identified both annotation challenges (ambiguous inputs, cross-category overlap, and inconsistencies) and inference limitations (semantic overlap, implicit meanings, and contextual misinterpretation). On external datasets, Flan-T5 models outperformed other LLMs (*P* < .001), and were more robust to demographic descriptors, while GPT-4.1 and Gemma3 often misclassified inputs as “None.” Additionally, GPT-4.1’s inference consumed ∼30× more energy than that of Flan-T5-xl.

**Discussion:**

Fine-tuned Flan-T5 models demonstrated strong classification performance, greater robustness to demographic attributes, and lower energy consumption, though challenges remained for subjective and underrepresented categories, reflecting both data imbalance and model limitations in implicit reasoning.

**Conclusion:**

LLM-based approaches show promise for real-time ART adherence monitoring, offering a scalable solution to individualized HIV care. Beyond performance, our findings highlight the importance of fairness and environmental sustainability in clinical AI development, with next steps focused on real-world validation through deployment in patient-facing digital tools.

## Background

Antiretroviral therapy (ART) has transformed HIV into a manageable chronic condition, substantially increasing the life expectancy of people with HIV (PWH).^1^^,^^2^ However, maintaining optimal ART adherence remains a persistent challenge. Evidence from a Canadian retrospective study found that nearly 20% of PWH had adherence rates below 85%.^3^ Similarly, a U.S. study^4^ and a multi-country survey^5^ reported that 40% and 25% of individuals, respectively, fell below 80% adherence.^6^^,^^7^ Suboptimal adherence not only leads to poorer clinical outcomes^8^ and viral resistance^9^ but also increases the risk of loss to follow-up^10^^,^^11^ and onward transmission.^12^

Adherence barriers are factors that interfere, whether temporarily or persistently, with a person’s ability to take medication as prescribed. Early identification is essential to enable timely intervention and prevent or limit treatment interruptions. Multiple clinical guidelines^13^^,^^14^ emphasize the importance of regularly assessing adherence barriers to promote personalized, patient-centered care. Yet, identifying these factors in routine clinical care remains difficult. Experiences of adherence may be discussed during medical consultations, but these are often constrained by limited time, competing demands, or patient discomfort discussing sensitive topics, and even physician unease in initiating such conversations.^15–18^ Patient-reported outcome measures (PROMs) provide a standardized alternative but are infrequently integrated into HIV care due to concerns about increased workload, patient motivation, and unfamiliarity with interpreting results.^19^^,^^20^ Retrospective approaches such as pharmacy refill data or electronic health records (EHR)^21^ can offer useful insights into ART adherence patterns. However, they are inherently reactive, typically identifying problems only after adherence has declined, without necessarily revealing the underlying reasons for missed doses or allowing for anticipation of emerging adherence challenges.^22^^,^^23^ Proactive strategies are therefore needed to detect early warning signs and intervene before significant lapses occur.

In recent years, mobile health (mHealth) tools—such as SMS/text messaging,^24–26^ patient portals,^27–30^ and chatbots^31–33^—have been explored to support ART adherence by enhancing patient engagement in care,^26^^,^^27^^,^^30^ improving communication,^25^^,^^33^ and facilitating self-management.^24^^,^^31^ These tools are perceived by PWH as acceptable and easy to use,^24^^,^^28–32^^,^^34^ with positive impacts on adherence documented in several studies.^24^^,^^28–30^^,^^34^ However, their effectiveness often declines over time.^24^^,^^34^ Research highlights the need for more comprehensive, personalized, and proactive content to sustain user engagement and address evolving needs.^24^^,^^31^^,^^32^^,^^34^ Interestingly, patients generate a large volume of messages during interactions with mHealth platforms, which contain rich yet mostly untapped information relevant to adherence monitoring. One promising strategy is to analyze these messages to triage emerging adherence problems and tailor interventions accordingly. The advent of large language models (LLMs) presents a viable opportunity to automatically detect ART adherence barriers from these unstructured data streams. Prior research has shown the potential of AI-based natural language processing (NLP) techniques to analyze patient messages for early detection of depression^35^ and mental health crises,^36^^,^^37^ both recognized as well-established barriers to ART adherence. Leveraging such approaches may enable earlier identification of patients at risk of nonadherence, facilitating timely and targeted interventions.

Despite growing interest in such applications, limited information exists on which models are best suited for processing unstructured patient messages. Model selection is critical in healthcare AI model development,^38^ where clinical foundation models—pretrained on biomedical or clinical records—are often assumed to outperform general-domain models following fine-tuning for downstream clinical NLP tasks.^39–41^ However, recent studies have challenged their added value in real-world settings.^42^ Despite strong performance on benchmark NLP tasks, these models may not generalize well to such informal, patient-facing tasks. This is particularly relevant for classification tasks involving patient-generated messages, which use everyday language that may not be well captured in clinical training data.^35^ In parallel, larger-scale LLMs such as GPT-4 have demonstrated robust performance on standardized exams (eg, with GPT-4 passing the United States Medical Licensing Examination (USMLE)^43^) and clinical knowledge in HIV care,^44^^,^^45^ but this does not guarantee their ability to identify nuanced adherence barriers from patient-generated messages. These gaps highlight the need to evaluate model suitability for identifying adherence challenges in real-world HIV care contexts.

Equally important is ensuring the fair and sustainable development and deployment of LLM-based models in HIV care. Responsible AI development needs to align with social and environmental values and minimize potential harms.^46^ For example, a key fairness concern is that LLMs may perpetuate racial and gender biases in healthcare,^47^ which is particularly troubling in contexts where intersecting stigma already disproportionately impacts marginalized communities.^48^^,^^49^ Without careful design and oversight, these technologies risk amplifying existing inequities and reinforcing the very stigma they should dismantle. Sustainability is another essential consideration,^50^ particularly as LLMs gain traction in healthcare and their environmental footprint raises growing concerns about the long-term viability of AI.^51^^,^^52^ Transparency must extend beyond publishing performance results to include energy consumption and carbon emissions to support more informed and targeted model development and deployment decisions. Assessing these key model features is critical to inform the development of LLM-based tools to support ART adherence and ensure their effective deployment in real-world healthcare settings.

## Objectives

The primary objective of this study was to develop and validate LLM-based triage models to automatically and proactively identify ART adherence challenges from patient-generated messages. Specifically, we aimed to (1) assess the comparative performance of fine-tuned general-domain versus clinical foundation models, alongside the GPT family and other open-source LLMs on adherence barrier and associated nonadherence risk classification informed by adherence level thresholds; (2) examine potential model biases related to racial and gender descriptors; and (3) assess the energy consumption and carbon footprints of model development and deployment.

## Methods

### Participatory design approach

Given the interdisciplinary scope and the patient-centered nature of the study, we adopted a participatory design approach.^53^ A co-construction committee was established at the outset of the study, comprising 3 PWH, 3 HIV care specialists (1 pharmacist, 2 research nurses), 1 medical resident, and 1 engineering researcher. The committee actively contributed to key stages of the project, including data annotation, model development, and validation. This collaborative approach ensured that the evolving tool reflected patient priorities and supported the development of a trustworthy healthcare AI solution.^54^^,^^55^

### Ethical considerations

This study received approval from the McGill University Health Center Research Ethics Board on August 10, 2023 (approval no. MP-37-2023-9333R).

### Conceptual framework and definitions

ART adherence barriers are complex and multifactorial. To guide our classification, we drew on the conceptual framework and content of the 7-item Interference-Score (I-Score) PROM—a stakeholder-informed tool developed to facilitate the identification and discussion of ART adherence barriers in routine HIV care.^56^ The I-Score captures the frequency of barriers within 7 domains and our barrier categories mirror these:

1. Thoughts & Feelings: this includes barriers related to acceptance of having HIV, emotions (feeling sad, anxious, etc.), medication-related knowledge and beliefs, or motivation to take medication.
2. Habits & Activities: this encompasses barriers tied to daily life (one’s schedule, priorities, etc.) or substance use (alcohol, drugs, or other substances).
3. Social Situation: this involves barriers due to personal relationships or the experience of stigma.
4. Economic Situation: this includes financial or housing-related barriers.
5. Medication: this includes barriers associated with the HIV medication, namely its side effects, instructions, or physical features (for example, pill size or taste).
6. Care: this refers to barriers due to issues with healthcare professionals, the clinic, the pharmacy, or payment of medication or care.
7. Health: this includes barriers attributed to lab test results, HIV symptoms, or overall health.

To inform the risk classification scheme, we defined adherence levels as a function of the number of nonadherent days within a 30-day period, based on clinical literature^5^^,^^6^^,^^57^ and committee input:

- Perfect adherence: No missed doses
- Optimal adherence: ≥95% and  <100% (ie, ≤1 missed dose)
- Near-optimal adherence:  <95% and ≥80% (ie, 2-5 missed doses, not occurring consecutively)
- Suboptimal adherence:  <80% (ie, ≥6 missed doses)

Overall, the 4-level risk classification scheme takes into account the severity and frequency of adherence-related challenges in the message.

1. High: Explicit mention of an adherence barrier that is currently causing, or is highly likely to lead to, suboptimal adherence.
2. Medium: Explicit mention of an adherence barrier that is currently causing, or is highly likely to lead to, near-optimal adherence.
3. Low: Explicit mention of an adherence barrier that is currently causing, or is highly likely to lead to, optimal but not perfect adherence.
4. None: No mention of adherence barriers, or explicit mention of a barrier with an explicit statement of no current or imminent adherence risk.

Table 1 illustrates how these classifications may translate into clinical triage actions across representative patient messages. Suggested responses reflect the clinical logic of each risk level; delivery mechanisms will vary by implementation platform.

**Table 1.** Illustrative examples of model inputs, outputs, and potential clinical triage actions.

Inputs	Outputs		Potential clinical response
	Barrier	Risk level	
And that might mean another 5 days of not taking my medications, cause I will not go back to the pharmacy until my next day off.	Care	High	Immediate alert to care provider (eg, via EHR flag); patient referred for clinical follow-up (eg, chatbot escalation, portal notification)
When I travel, sometimes I’m surrounded by several people, sometimes, like on those days, I didn’t take it.	Social situation	Medium	Barrier-targeted education; high-frequency reminders and proactive check-ins (eg, chatbot or portal messaging)
I’m just curious because I’m going to a party tonight probably with some drinking, and I’m wondering if I can still take my meds, like what is the best action? :)	Habits & Activities	Low	Single barrier-targeted education; periodic check-in
I didn’t see any difference, because between taking a small tablet and a bigger one at the same time, or taking just one, it doesn’t make much difference.	Medication	None	Routine care continues; no immediate action required
Examples are drawn from the annotated dataset. Suggested triage actions reflect the clinical logic of the classification output; specific delivery mechanisms will vary by implementation platform (eg, chatbot, patient portal, EHR).			

### Data

We compiled a primary de-identified dataset from multiple sources:

1)  MARVIN Training corpus (MT): MARVIN is an AI-based chatbot developed since 2020 that provides self-management support to PWH through English and French text-based conversations.^31^ We included its English training corpus, co-developed with patient partners, healthcare providers, and developers. This corpus covers 113 topics, including:
  •  ART guidance (eg, timing, dosing, drug interactions, and side effects).
  •  ART management during travel.
  • Common HIV-related knowledge (eg, symptoms, transmission, prevention, and vaccination).
2)  MARVIN User conversations (MU): Conversation records collected from 1243 MARVIN users between 2021 and 2024 (1075 in English; 168 in French).
3)  I-Score study interviews (ISCORE): We included 27 interview transcripts (15 English, 12 French) from the I-Score development study conducted with PWH in Canada between 2016 and 2017. These interviews primarily explored participants’ real-life experiences with ART adherence, including the challenges they faced or anticipated in maintaining treatment.

 Two external validation datasets were also assembled:

1. Online Forum messages (OF): A collection of 359 English-language thread messages related to ART were randomly selected from the Reddit r/HIV,^58^ r/hivaids forums,^59^ and the POZ community forum,^60^ using the keywords “medication” and “ART treatment” from 2024 onwards.
2. Portail VIH/SIDA du Québec messages (PVSQ): A set of 320 anonymous, user-submitted messages (7 in English; 313 in French) collected between October 2023 and May 2025 through the Q&A messaging portal of the Portail VIH/SIDA du Québec.^61^ This community organization provides information and support related to HIV and other sexually transmitted and blood-borne infections (STBBIs). The collected messages covered topics ranging from HIV and STBBIs prevention, treatment, transmission, and daily life with these conditions.

These sources were selected for their relevance to real-world HIV care, their digital and conversational nature, and the richness of language used to describe adherence-related experiences, making them well-suited for developing triage models applied to patient-generated messages. Given the multifactorial nature of ART adherence challenges, individual messages often contained descriptions of multiple and intertwined potential barriers. To ensure sentence-level annotation and reliable classification, all data were split into sentences using the *syntok* sentence segmenter Python library.^62^ Preprocessing steps included translation of French content to English, removal of HTML tags and URLs, and de-duplication.

### Data annotation

Two sentence-level, multiclass classification tasks were defined. Each sentence was annotated with one label per task, reflecting its most relevant barrier type and risk level (see Table 1 for illustrative examples):

- Task I: ART adherence barrier classification—Sentences were categorized into 1 of 7 barrier types by referencing the I-Score PROM. An eighth category, *None*, was used if the sentence did not mention any adherence barriers.
- Task II: Triage risk level classification—Sentences were categorized into 1 of 4 nonadherence risk levels.

**Figure 1.**
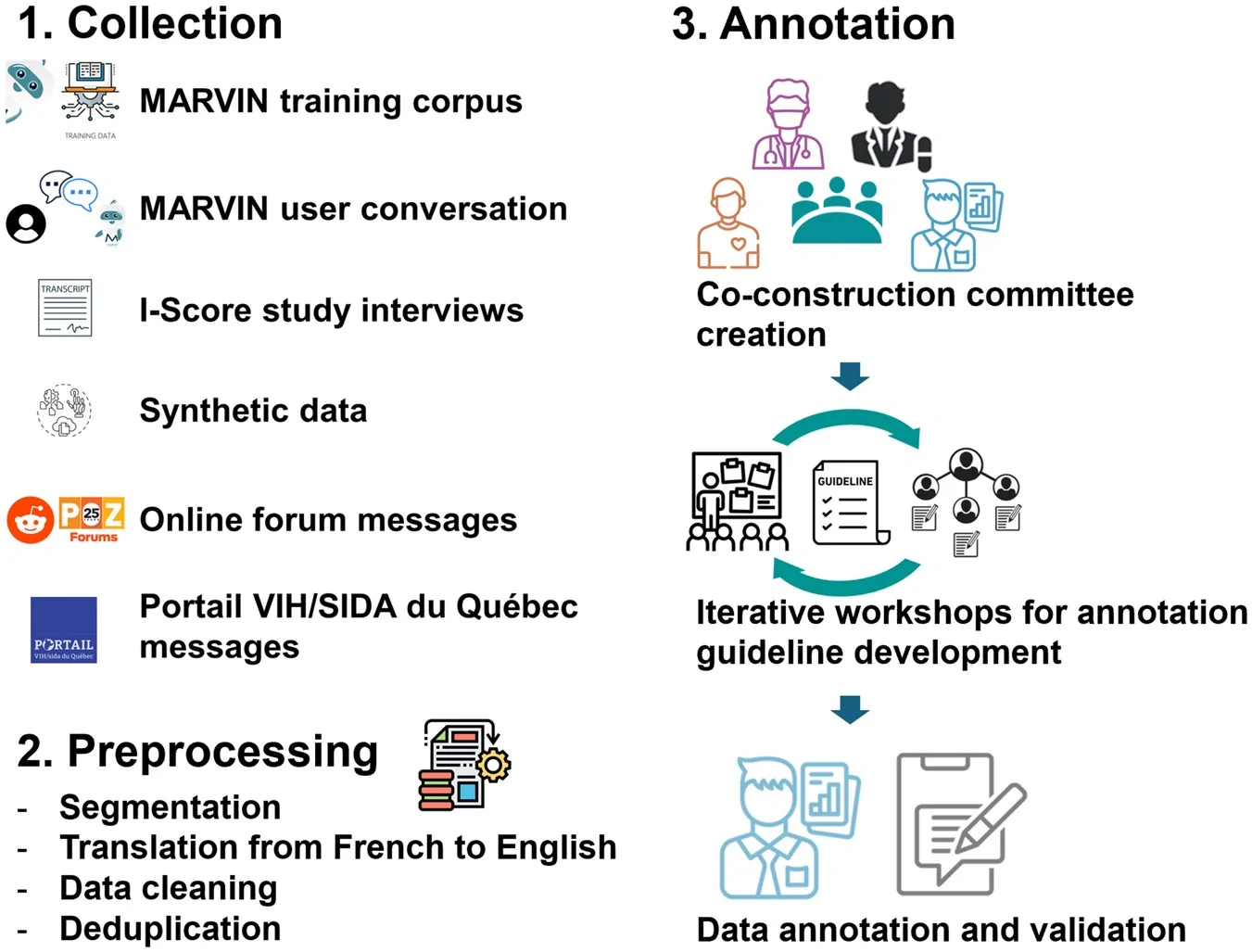
Data preparation and annotation process with the co-construction committee.

The data annotation process is illustrated in Figure 1. Through three 90-minute workshops, the co-construction committee iteratively developed and refined the annotation guideline. Between workshops, the engineering researcher and the medical resident independently annotated 10% of the dataset following the evolving guideline. Ambiguous cases were discussed during workshops, and the guideline was updated accordingly. After confirming reliability, one researcher completed the full dataset annotation using the finalized guideline (see Supplementary File 1).

### Data augmentation

In the collected training data, sentences related to *Economic Situation* accounted for only 1.2% of the total (162/13780), while sentences related to *None* accounted for 38.6% (5319/13780). To address class imbalance in the training dataset, we applied synthetic data augmentation, a strategy shown to improve model performance under such conditions.^63^ Using 6 LLMs, we generated candidate sentences for each barrier category and risk level. Following manual review for quality and topic relevance, only validated sentences were included in the final training dataset. Details of the LLMs and prompting procedures are provided in the Supplementary File 2.

Table 2 presents the characteristics of the final curated datasets, including sentence distributions and sentence length statistics. The primary dataset (MT + MU + ISCORE) contained 13 780 annotated sentences; the synthetic dataset included 700 validated sentences; and the external validation dataset (OF + PVSQ) had 1000 annotated instances. Inter-annotator agreement was assessed using Cohen’s Kappa coefficient, yielding scores of 0.83 for barrier annotation and 0.71 for risk level, indicating almost perfect and substantial agreement, respectively.^64^

### Model development

The primary dataset was randomly split into training (80%), validation (10%), and test (10%) sets. The OF + PVSQ datasets, along with the test split, were held out for external assessment throughout model development. The synthetic dataset was incorporated into the training set to address class imbalance.

Model development focused on the effectiveness of different LLMs for classifying ART adherence barriers and associated risk levels. We fine-tuned general-domain language models, including BERT-base^65^ and Flan-T5 (-small, -base, -large, and -xl).^66^ We also fine-tuned clinical foundation models from both families: Bio-BERT,^39^ BioClinicalBERT,^41^ and Clinical-T5(-base and -large).^40^

All model architectures were adapted for multiclass sequence classification. Cross-entropy loss was used for all models. To reduce computational demands, Flan-T5-large and -xl models were fine-tuned using parameter-efficient tuning via low-rank adaptation (LoRA).^67^ Class weighting was applied during training to mitigate bias toward the majority class. Model hyperparameters are provided in the Supplementary File 2.

### Model assessment

All models were monitored on the validation split during training, with final performance assessed on the held-out test split and external validation datasets. Multiclass prediction performance was measured using the Macro-F1 score, which provides a balanced evaluation of precision (positive predictive value) and recall (sensitivity) across all classes. Table 3 provides a clinical interpretation of these metrics.

**Table 2.** Characteristics of the development and validation datasets.

Dataset overview	Barrier								Risk level			
	Thoughts & Feelings	Habits & Activities	Social Situation	Economic Situation	Medication	Care	Health	None	High	Medium	Low	None
**Development**												
MT (*n* = 3874)	302 (7.8%)	541 (14.0%)	91 (2.3%)	0 (0.0%)	611 (15.8%)	56 (1.4%)	823 (21.2%)	1450 (37.4%)	140 (3.6%)	687 (17.7%)	1068 (27.6%)	1979 (51.1%)
MU (*n* = 3721)	205 (5.5%)	802 (21.6%)	137 (3.7%)	5 (0.1%)	612 (16.4%)	29 (0.8%)	447 (12.0%)	1484 (39.9%)	183 (4.9%)	653 (17.5%)	1120 (30.1%)	1765 (47.4%)
ISCORE (*n* = 6185)	1047 (16.9%)	742 (12.0%)	444 (7.2%)	157 (2.5%)	522 (8.4%)	532 (8.6%)	356 (5.8%)	2385 (38.6%)	217 (3.5%)	1313 (21.2%)	819 (13.2%)	3836 (62.0%)
MT + MU + ISCORE (*n* = 13 780)	1554 (11.3%)	2085 (15.1%)	672 (4.9%)	162 (1.2%)	1745 (12.7%)	617 (4.5%)	1626 (11.8%)	5319 (38.6%)	540 (3.9%)	2653 (19.3%)	3007 (21.8%)	7580 (55.0%)
Train (*n* = 11 023)	1243 (11.3%)	1668 (15.1%)	538 (4.9%)	129 (1.2%)	1395 (12.7%)	494 (4.5%)	1301 (11.8%)	4255 (38.6%)	440 (4.0%)	1902 (17.3%)	2596 (23.6%)	6085 (55.2%)
Valid (*n* = 1377)	167 (12.1%)	215 (15.6%)	60 (4.4%)	19 (1.4%)	183 (13.3%)	54 (3.9%)	155 (11.3%)	524 (38.1%)	49 (3.6%)	400 (29.0%)	198 (14.4%)	730 (53.0%)
Test (*n* = 1380)	144 (10.4%)	202 (14.6%)	74 (5.4%)	14 (1.0%)	167 (12.1%)	69 (5.0%)	170 (12.3%)	540 (39.1%)	51 (3.7%)	351 (25.4%)	213 (15.4%)	765 (55.4%)
SD (*n* = 700)	79 (11.3%)	77 (11.0%)	81 (11.6%)	259 (37.0%)	53 (7.6%)	91 (13.0%)	60 (8.6%)	0 (0.0%)	155 (22.1%)	267 (38.1%)	172 (24.6%)	106 (15.1%)
**Validation**												
PVSQ (*n* = 641)	41 (6.4%)	16 (2.5%)	24 (3.7%)	11 (1.7%)	16 (2.5%)	81 (12.6%)	300 (46.8%)	152 (23.7%)	53 (8.3%)	137 (21.4%)	201 (31.4%)	250 (39.0%)
OF (RDT + POZ, *n* = 359)	56 (15.6%)	52 (14.5%)	32 (8.9%)	43 (12.0%)	65 (18.1%)	42 (11.7%)	58 (16.2%)	11 (3.1%)	36 (10.0%)	205 (57.1%)	74 (20.6%)	44 (12.3%)

All values are presented as *n* (%). The primary dataset (MT + MU + ISCORE) was randomly divided into training (80%), validation (10%), and test (10%) subsets. The synthetic dataset (SD) was incorporated into the training set, resulting in a final training sample size of *n* = 11 723.

Abbreviations: ISCORE, I-Score study interviews; MT, MARVIN Training corpus; MU, MARVIN User conversation; OF, online forum; POZ, POZ community forum; PVSQ, Portail VIH/SIDA Québec; RDT, Reddit forum; SD, synthetic data.

**Table 3.** Clinical interpretation of model performance metrics.

Term	Definition
Macro-F1	Summary metric balancing precision and recall equally across all categories (range = 0-1: higher is better)
Precision	Proportion of flagged messages truly containing a barrier or risk level signal (analogous to positive predictive value; range = 0-1: higher is better)
Recall	Proportion of true barrier/risk level messages correctly detected (analogous to sensitivity; range = 0-1: higher is better)
Binary F1	Discrimination between presence and absence of any barrier or risk level signal, collapsing all positive categories against None

The F1 score is defined as the harmonic mean of precision and recall:


$$F1=\frac{2\times \mathrm{Precision}\times \mathrm{Recall}}{\mathrm{Precision}\;+\;\mathrm{Recall}},\;\mathrm{where}\;\mathrm{Precision}=\frac{\mathrm{TP}}{\mathrm{TP}\;+\;\mathrm{FP}},\;\mathrm{Recall}=\frac{\mathrm{TP}}{\mathrm{TP}\;+\;\mathrm{FN}}.$$

The Macro-F1 score is computed as the unweighted average of the F1 scores for all *N* classes:


$$\mathrm{Macro}-F1=\frac{1}{N}\sum_{i=1}^{N}{F1}_{i},$$

where TP = true positives, FP = false positives, and FN = false negatives.

For the best-performing models, a binary task reformulation was additionally performed to provide a clinically intuitive estimate of the model’s ability to detect any adherence concern, analogous to a screening test’s sensitivity. All detected barrier or risk levels were grouped together and compared against the *None* category, with the F1 score used to quantify performance.

### Error analysis

An inductive error analysis was conducted on misclassified examples from the primary test split dataset using the best-performing model to support model explainability and identify common error patterns. To ensure credibility and reliability, results were debriefed with the co-construction committee at the fourth workshop.

### Comparison with other LLMs

We assessed the performance of proprietary GPT family LLMs (GPT-4.1-2025-04-14, -mini, and -nano) and open-source models (LLaMA3.2-3B, DeepSeek-R1-7B, Qwen3-8B, Gemma3-12B, and Mistral-7B) on the external validation datasets. Their results were compared with those of our best-performing fine-tuned models.

All models were prompted using both zero-shot and 5-shot chain-of-thought (CoT) approaches. Test inputs were embedded within a standardized prompt template (see Supplementary File 2), instructing the model to perform multiclass classification and return predictions in structured JSON format. For the 5-shot setting, examples were randomly sampled from the training split. To ensure reproducibility, the temperature parameter was fixed at zero for all models.

### Assessment of model bias and environmental footprint

We evaluated potential model bias related to race and gender by calculating the prediction mismatch rate after introducing identity descriptors into originally labeled sentences. Following established approaches,^68^^,^^69^ we focused on sentences originally categorized as containing barriers or risk levels (ie, not labeled *None*), since false positives carry relatively low risk in triage classification.^37^^,^^70^

**Figure 2.**
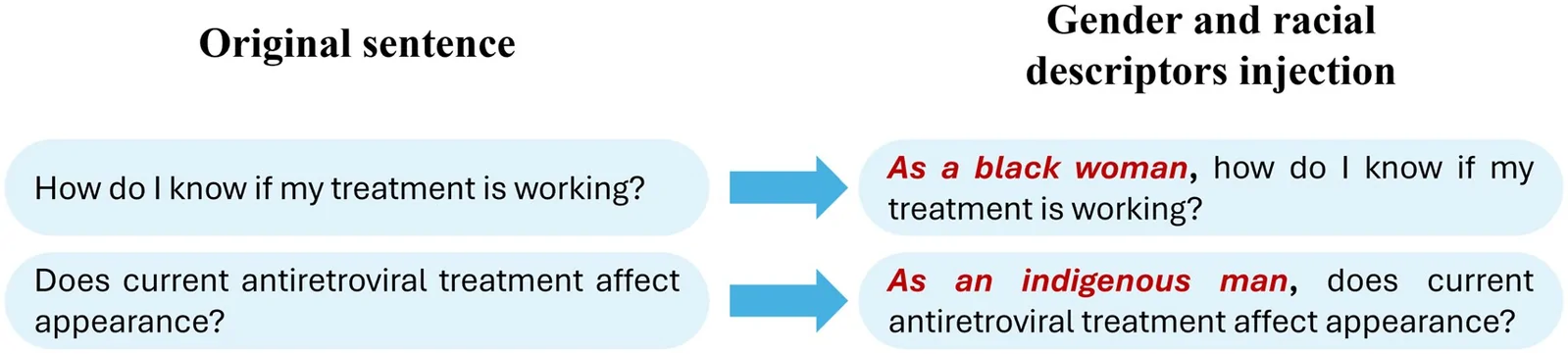
Examples of injected gender and racial descriptors used to assess model fairness.

Using GPT-4.1-mini, combined gender and racial descriptors (eg, *Asian woman*, *Black man*, and *Latino trans person*) were randomly inserted into selected sentences from the de-identified external validation set (see Figure 2 for examples). The following descriptors were applied:

- Gender: woman, man, and trans
- Race: Asian, Black, Indigenous, Latino, and White

After manual validation to ensure semantic coherence, model inference was performed on 837 modified sentences, and we calculated the prediction mismatch. Full prompt details are available in the Supplementary File 2.

To assess the environmental sustainability of our models, energy consumption and carbon footprint were estimated during both fine-tuning and inference using the *CodeCarbon*^71^ Python package. Both metrics were tracked for each fine-tuned model to compare development-related environmental impact. For inference, we compared our best-performing model with the other LLMs based on published estimates. All values were normalized per 1000 inferences, and the host country of the cloud computing infrastructure for fine-tuning and inference was reported to account for regional variation in carbon emissions.

### Statistical analysis

Model performance was evaluated by estimating the mean Macro-F1 and corresponding 95% confidence intervals (CIs) using non-parametric bootstrap sampling with the percentile method on the test set and external validation datasets. The bootstrap sample size matched each dataset, with sampling performed with replacement. A total of 300 bootstrap iterations were conducted to ensure that the standard error of the CI limits remained below 0.01. The same procedure was used to estimate CIs for the binary classification F1 scores.

Pairwise comparisons of Macro-F1 means between general-domain language models and clinical foundation models of matched architecture and size were performed using the Mann-Whitney U test. The best-performing fine-tuned models were compared with other LLMs using the same test, and Bonferroni correction was applied for multiple comparisons.

Mismatch rates in barrier and risk level classification, before and after the injection of racial and gender descriptors, were compared using chi-square tests for each subgroup.

A 2-sided significance level of 5% was applied for all statistical tests. Analyses were conducted using the Python *SciPy*^72^ package.

## Results

### Model performance on primary test split

Table 4 summarizes fine-tuned model performance for both classification tasks using the primary dataset test split.

**Table 4.** Fine-tuned model performance on test split–primary dataset.

Task I—Barrier prediction												
Model	Parameters (Total/Tuned)	Mean Macro-F1 (95% CI)	ΔMacro-F1	*P* value	Thoughts & Feelings (F1)	Habits & Activities (F1)	Social Situation (F1)	Economic Situation (F1)	Medication (F1)	Care (F1)	Health (F1)	None (F1)
**BERT-base**	110M/110M	**0.85 (0.82-0.87)**			0.70	0.86	0.77	**0.83**	0.89	0.89	0.92	**0.92**
BioBERT	110M/110M	0.81 (0.78-0.84)	−0.04	<.001	0.65	0.86	0.70	0.76	0.88	0.86	0.91	0.91
BioClinicalBERT	110M/110M	0.83 (0.80-0.85)	−0.02	<.001	0.67	0.85	0.76	**0.83**	0.88	0.86	0.89	0.91
Flan-T5-small	77M/77M	0.76 (0.72-0.79)			0.57	0.81	0.66	0.71	0.83	0.78	0.87	0.87
Flan-T5-base	248M/248M	0.83 (0.80-0.86)			0.72	**0.87**	0.78	0.69	0.89	0.85	**0.94**	**0.92**
Clinical-T5-base	248M/248M	0.77 (0.73-0.80)	−0.06	<.001	0.61	0.80	0.67	0.67	0.86	0.77	0.91	0.86
Flan-T5-large	760M/4.7M	0.83 (0.80-0.86)			**0.73**	**0.87**	0.78	0.62	**0.90**	**0.92**	0.92	**0.92**
Clinical-T5-large	760M/4.7M	0.72 (0.69-0.76)	−0.11	<.001	0.57	0.75	0.60	0.56	0.81	0.79	0.87	0.86
Flan-T5-xl	3B/9.4M	0.83 (0.80-0.87)			0.72	**0.87**	**0.79**	0.71	0.88	0.87	0.91	**0.92**

Task II—Risk level prediction								
Model	Parameters (Total/Tuned)	Mean Macro F1 (95% CI)	ΔMacro-F1	*P* value	High (F1)	Medium (F1)	Low (F1)	None (F1)
**BERT-base**	110M/110M	**0.80 (0.76-0.82)**			0.69	**0.81**	0.78	**0.91**
BioBERT	110M/110M	0.79 (0.76-0.82)	−0.01	<.001	0.73	0.80	0.75	0.89
BioClinicalBERT	110M/110M	0.77 (0.73-0.80)	−0.03	<.001	0.65	0.80	0.73	0.89
Flan-T5-small	77M/77M	0.64 (0.60-0.67)			0.48	0.69	0.56	0.83
**Flan-T5-base**	248M/248M	**0.80 (0.77-0.83)**			0.70	**0.81**	**0.80**	**0.91**
Clinical-T5-base	248M/248M	0.72 (0.68-0.75)	−0.08	<.001	0.56	0.75	0.67	0.88
Flan-T5-large	760M/4.7M	0.79 (0.75-0.82)			0.67	**0.81**	0.76	**0.91**
Clinical-T5-large	760M/4.7M	0.68 (0.64-0.71)	−0.10	<.001	0.59	0.71	0.57	0.85
Flan-T5-xl	3B/9.4M	0.79 (0.76-0.82)			**0.74**	0.79	0.74	0.90

The 95% CIs for Macro-F1 scores were calculated using 300 bootstrap samples with replacement on 3 distinct datasets, ensuring a SE of the CI limits below 0.01. The observed SE interval limit was 0.0051. ΔMacro-F1 represents the Macro-F1 difference between fine-tuned general-domain models and fine-tuned clinical foundation models of the same architecture (eg, BERT vs BioBERT). Bold values indicate the highest performance for each metric, including mean Macro-F1 and category-specific F1 scores; ties are also shown in bold. *P* values were calculated using the Mann-Whitney U test.

Abbreviations: CI, confidence interval; SE, standard error.

For barrier prediction (Task I), the best-performing model was BERT-base, achieving a Macro-F1 score of 0.85 (95% CI, 0.82-0.87) and the highest F1 scores for *Economic Situation* and *None*.

For risk level prediction (Task II), the best-performing models were BERT-base and Flan-T5-base, both reaching a Macro-F1 score of 0.80 (BERT-base: 95% CI, 0.76-0.82; Flan-T5-base: 95% CI, 0.77-0.83). Notably, Flan-T5-base achieved the highest F1 scores in 3 of the 4 risk categories (*Medium*, *Low*, and *None*).

Except for Flan-T5-small, all other general-domain models performed comparably after fine-tuning, with Macro-F1 differences within 0.02 of the best-performing models across both tasks.

For both tasks, model performance varied across categories. The *None* category showed the highest performance. In barrier prediction, the *Medication* and *Health* barrier categories also performed well, followed by *Care* and *Habits & Activities*. The remaining 3 categories (*Thoughts & Feelings*, *Social Situation*, and *Economic Situation*) showed more variable performance across models. For risk level prediction, the *Medium* category performed second best after *None*, followed by *Low*, while *High* performed moderately across models.

### Comparison of general-domain and clinical foundation models

Across both tasks, general-domain language models significantly outperformed clinical foundation models following fine-tuning (*P* < .001). The performance gap increased with model size, as reflected by increasing Δ Macro-F1 values (−0.01 to −0.11).

When comparing model performance across categories for each task, general and clinical T5-based models performed similarly in *Economic Situation*, *Medication*, *Health*, and *None* categories in Task I, while larger F1 score gaps were observed in the remaining 4 categories. For BERT-based models, larger gaps in F1 scores were observed in the *Thoughts & Feelings, Social Situation,* and *Economic Situation* categories compared with the other categories. For Task II, all 4 model pairs showed large F1 score differences in the *High* and *Low* categories, while in the *Medium* and *None* categories they had more modest differences.

### External validation performance

Table 5 presents fine-tuned model performance on the external validation datasets. Performance generally scaled with model size on external data.

**Table 5.** Fine-tuned model performance on external validation dataset.

Task I—Barrier prediction												
Model	Parameters (Total/Tuned)	Mean Macro F1 (95% CI)	ΔMacro-F1	*P* value	Thoughts & Feelings (F1)	Habits & Activities (F1)	Social Situation (F1)	Economic Situation (F1)	Medication (F1)	Care (F1)	Health (F1)	None (F1)
BERT-base	110M/110M	0.64 (0.61-0.67)			0.58	0.66	0.63	0.70	0.49	0.57	0.77	0.70
BioBERT	110M/110M	0.65 (0.62-0.68)	+ 0.01	<.001	0.57	0.66	0.60	0.70	0.63	0.60	0.81	0.67
BioClinicalBERT	110M/110M	0.63 (0.60-0.67)	−0.01	.003	0.54	0.66	0.59	**0.73**	0.65	0.50	0.77	0.64
Flan-T5-small	77M/77M	0.56 (0.53-0.60)			0.38	0.55	0.53	0.64	0.46	0.56	0.73	0.67
Flan-T5-base	248M/248M	0.68 (0.64-0.70)			0.60	0.72	0.62	0.70	0.63	0.62	0.80	0.73
Clinical-T5-base	248M/248M	0.58 (0.55-0.62)	−0.08	<.001	0.48	0.63	0.45	0.60	0.61	0.53	0.78	0.61
Flan-T5-large	760M/4.7M	0.68 (0.65-0.72)			0.55	**0.76**	0.61	0.69	0.71	**0.64**	**0.83**	0.70
Clinical-T5-large	760M/4.7M	0.55 (0.52-0.59)	−0.13	<.001	0.37	0.53	0.43	0.57	0.60	0.53	0.75	0.67
**Flan-T5-xl**	3B/9.4M	**0.71 (0.68-0.74)**			**0.69**	**0.76**	**0.66**	0.67	**0.74**	0.58	**0.83**	**0.74**

Task II—Risk level prediction								
Model	Parameters (Total/Tuned)	Mean Macro F1 (95% CI)	ΔMacro-F1	*P* value	High (F1)	Medium (F1)	Low (F1)	None (F1)
BERT-base	110M/110M	0.51 (0.47-0.54)			0.36	0.55	0.47	0.65
BioBERT	110M/110M	0.52 (0.48-0.55)	+ 0.01	<.001	0.38	0.59	0.46	0.64
BioClinicalBERT	110M/110M	0.52 (0.48-0.55)	+ 0.01	<.001	0.39	0.57	0.49	0.63
Flan-T5-small	77M/77M	0.42 (0.38-0.45)			0.30	0.42	0.41	0.54
Flan-T5-base	248M/248M	0.56 (0.53-0.60)			0.41	0.63	0.52	**0.69**
Clinical-T5-base	248M/248M	0.48 (0.45-0.51)	−0.08	<.001	0.33	0.53	0.48	0.58
**Flan-T5-large**	760M/4.7M	**0.57 (0.53-0.60)**			0.42	**0.65**	**0.55**	0.65
Clinical-T5-large	760M/4.7M	0.45 (0.42-0.49)	−0.12	<.001	0.37	0.43	0.46	0.56
Flan-T5-xl	3B/9.4M	0.56 (0.52-0.60)			**0.43**	0.62	0.54	0.65

The 95% CIs for Macro-F1 scores were calculated using 300 bootstrap samples with replacement on 3 distinct datasets, ensuring a SE of the CI limits below 0.01. The observed SE interval limit was 0.0044. ΔMacro-F1 represents the Macro-F1 difference between fine-tuned general-domain models and fine-tuned clinical foundation models of the same architecture (eg, BERT vs BioBERT). Bold values indicate the highest performance for each metric, including mean Macro-F1 and category-specific F1 scores; ties are also shown in bold. *P* values were calculated using the Mann-Whitney U test.

Abbreviations: CI, confidence interval; SE, standard error.

In Task I, Flan-T5-xl achieved the best overall Macro-F1 score of 0.71, ranking first in 6 of 8 barrier categories. For Task II, Flan-T5-large achieved the highest Macro-F1 score of 0.57, with top F1 score performance in the *Medium* and *Low* categories. Based on consistent results across both internal and external datasets, Flan-T5-xl and Flan-T5-large were identified as the best-performing models for Task I and Task II, respectively.

On external datasets, model performance declined by approximately 15% for barrier classification and 30% for risk level prediction, with mean Macro-F1 scores decreasing from 0.83 to 0.71 and from 0.80 to 0.57, respectively, compared to the primary test split.

Clinical-T5 models continued to underperform relative to Flan-T5 models (*P* < .001). In contrast, BioBERT slightly outperformed BERT-base in barrier detection, and both BioBERT and BioClinicalBERT achieved marginally better performance in risk level prediction (ΔMacro-F1= + 0.01, *P* < .001).

### Binary task reformulation analysis

Binary task reformulation analysis results for our best-performing fine-tuned models are provided in Table S2 in the Supplementary File 2. On the primary test split, Flan-T5-xl achieved an overall F1 score of 0.94 (95% CI, 0.93-0.95) for Task I, with F1 = 0.95 for detecting barrier-related sentences. For Task II, Flan-T5-large achieved an overall F1 of 0.90 (95% CI, 0.89-0.92), with F1 = 0.89 for identifying sentences indicating adherence risk.

On the external validation datasets, performance declined across both tasks. For Task I, Flan-T5-xl achieved an F1 score of 0.85 (95% CI, 0.82-0.88), with strong detection of barrier-related sentences (F1 = 0.96) but reduced performance on *None* sentences (F1 = 0.74). For Task II, Flan-T5-large achieved a F1 of 0.76 (95% CI, 0.72-0.78), with F1 = 0.86 for elevated adherence risk and F1 = 0.65 for non-risk sentences.

### Error analysis

The primary error patterns observed across both tasks are detailed in Table S3 in the Supplementary File 2 with examples. Inductive analysis revealed 6 distinct error types, stemming from either the human annotation process or model inference limitations:

Human annotation-related errors:

- Ambiguous or vague inputs: Short sentences that lack sufficient semantic detail, leading to multiple possible annotations.
- Cross-category cases: Sentences reflecting features of multiple predefined categories, complicating exclusive labeling.
- Inconsistent or incorrect annotations: Sentences inaccurately labeled due to human error during the annotation process.

Model inference-related errors:

- Semantic overlap: Misclassification due to conceptual overlap, often driven by over-reliance on shared vocabulary (eg, classifying sentences containing “holiday” as *Habits & Activities*, when it referred to taking a drug holiday).
- Implicit expression: Failure to detect implied meanings or pragmatic intent, limiting performance on socially or emotionally complex sentences.
- Contextual misinterpretation: Errors caused by information present in the broader message that was inaccessible at the sentence level.

The most common discrepancies between ground-truth and best-performing model prediction for each task are presented in Table S5 in the Supplementary File 2.

### Comparison with other LLMs on external validation datasets

Table 6 summarizes the results of the best-performing fine-tuned model for each task in comparison with the GPT family and other open-source LLMs on the external validation datasets.

**Table 6.** Best-performing fine-tuned model vs GPT & other open-source LLMs—performance on external validation dataset.

Task I—Barrier prediction												
Model	Parameters (Total/Tuned)	Mean Macro F1 (95% CI)	ΔMacro-F1	*P* value	Thoughts & Feelings (F1)	Habits & Activities (F1)	Social Situation (F1)	Economic Situation (F1)	Medication (F1)	Care (F1)	Health (F1)	None (F1)
**Flan-T5-xl**	3B/9.4M	**0.71 (0.68-0.74)**			**0.69**	**0.76**	**0.66**	0.67	**0.74**	0.58	**0.83**	**0.74**
**Five-shot**	Total parameters											
**GPT-4.1**	N/A	**0.62 (0.59-0.65)**	−0.09	<.001	0.54	0.70	0.63	0.66	0.73	0.64	0.57	0.51
GPT-4.1-mini	N/A	0.59 (0.56-0.63)	−0.12	<.001	0.52	0.60	0.61	0.76	0.65	0.64	0.51	0.47
GPT-4.1-nano	N/A	0.60 (0.57-0.63)	−0.11	<.001	0.45	0.66	0.53	0.75	0.66	**0.71**	0.58	0.49
Gemma3	12B	0.59 (0.56-0.63)	−0.12	<.001	0.51	0.42	0.57	0.74	0.65	0.58	0.74	0.55
Qwen3	8B	0.60 (0.57-0.63)	−0.11	<.001	0.52	0.62	0.63	**0.83**	0.67	0.60	0.50	0.43
Mistral	7B	0.58 (0.54-0.61)	−0.13	<.001	0.52	0.54	0.52	0.74	0.55	0.59	0.65	0.49
DeepSeek-R1	7B	0.45 (0.42-0.49)	−0.26	<.001	0.39	0.46	0.26	0.70	0.46	0.37	0.52	0.49
LLaMA3.2	3B	0.51 (0.48-0.55)	−0.20	<.001	0.48	0.47	0.37	0.66	0.51	0.43	0.67	0.53

Task II—Risk level prediction								
Model	Parameters (Total/Tuned)	Mean Macro F1 (95% CI)	ΔMacro-F1	*P* value	High (F1)	Medium (F1)	Low (F1)	None (F1)
**Flan-T5-large**	760M/4.7M	**0.57 (0.53-0.60)**			0.42	**0.65**	**0.55**	0.65
**Five-shot**	Total parameters							
GPT-4.1	N/A	0.43 (0.40-0.47)	−0.14	<.001	**0.50**	0.13	0.42	**0.67**
GPT-4.1-mini	N/A	0.42 (0.39-0.45)	−0.15	<.001	0.47	0.21	0.37	0.63
GPT-4.1-nano	N/A	0.43 (0.40-0.47)	−0.14	<.001	0.47	0.40	0.23	0.62
Gemma3	12B	0.47 (0.44-0.51)	−0.10	<.001	0.48	0.34	0.44	0.64
Qwen3	8B	0.33 (0.30-0.37)	−0.24	<.001	0.42	0.13	0.21	0.57
Mistral	7B	0.38 (0.35-0.41)	−0.19	<.001	0.38	0.33	0.21	0.60
DeepSeek-R1	7B	0.35 (0.31-0.38)	−0.22	<.001	0.29	0.27	0.28	0.55
LLaMA3.2	3B	0.28 (0.26-0.31)	−0.29	<.001	0.24	0.07	0.24	0.59
**Zero-shot**								
**Gemma3**	12B	**0.50 (0.46-0.54)**	−0.07	<.001	0.39	0.58	0.42	0.62

Total parameters of the GPT4 family models were not publicly available. The 95% CIs for Macro-F1 scores were calculated using 300 bootstrap samples with replacement on 3 distinct datasets, ensuring a SE of the CI limits below 0.01. The observed SE interval limit was 0.0039. ΔMacro-F1 represents the Macro-F1 difference between best-performing fine-tuned general-domain models and prompted GPT and other open-source LLMs. Bold values indicate the best performance for each metric among other LLMs, including mean Macro-F1 and category-specific F1 scores; ties are also shown in bold. *P* values were calculated using the Mann-Whitney U test, with Bonferroni correction applied for multiple comparisons (adjusted significance threshold: *P* < .00625, based on adjusted α = α/m, where α = 0.05, m = 8 (number of tests, 8 models)). Zero-shot results are provided in Table S4 in the Supplementary File 2, except Gemma3, shown here as the top performer for risk stratification.

Abbreviations: CI, confidence interval; N/A, not applicable; SE, standard error.

For Task I, Flan-T5-xl outperformed GPT-4.1 with 5-shot CoT prompting by an overall Δ Macro-F1 = −0.09 (*P* < .001), achieving the highest F1 scores in 6 of 8 barrier categories. For Task II, Flan-T5-large outperformed Gemma3-12B with zero-shot CoT prompting with an overall Δ Macro-F1 = −0.07 (*P* < .001), achieving the highest F1 scores for the *Medium* and *Low* risk levels. Interestingly, 5-shot GPT-4.1 received the best score of 0.50 for identifying a *High* risk level.

Across the remaining models, 5-shot CoT prompting generally improved performance over zero-shot prompting for Task I, while both prompting strategies yielded comparable results for Task II.

### Assessment of model bias and environmental footprint

**Figure 3.**
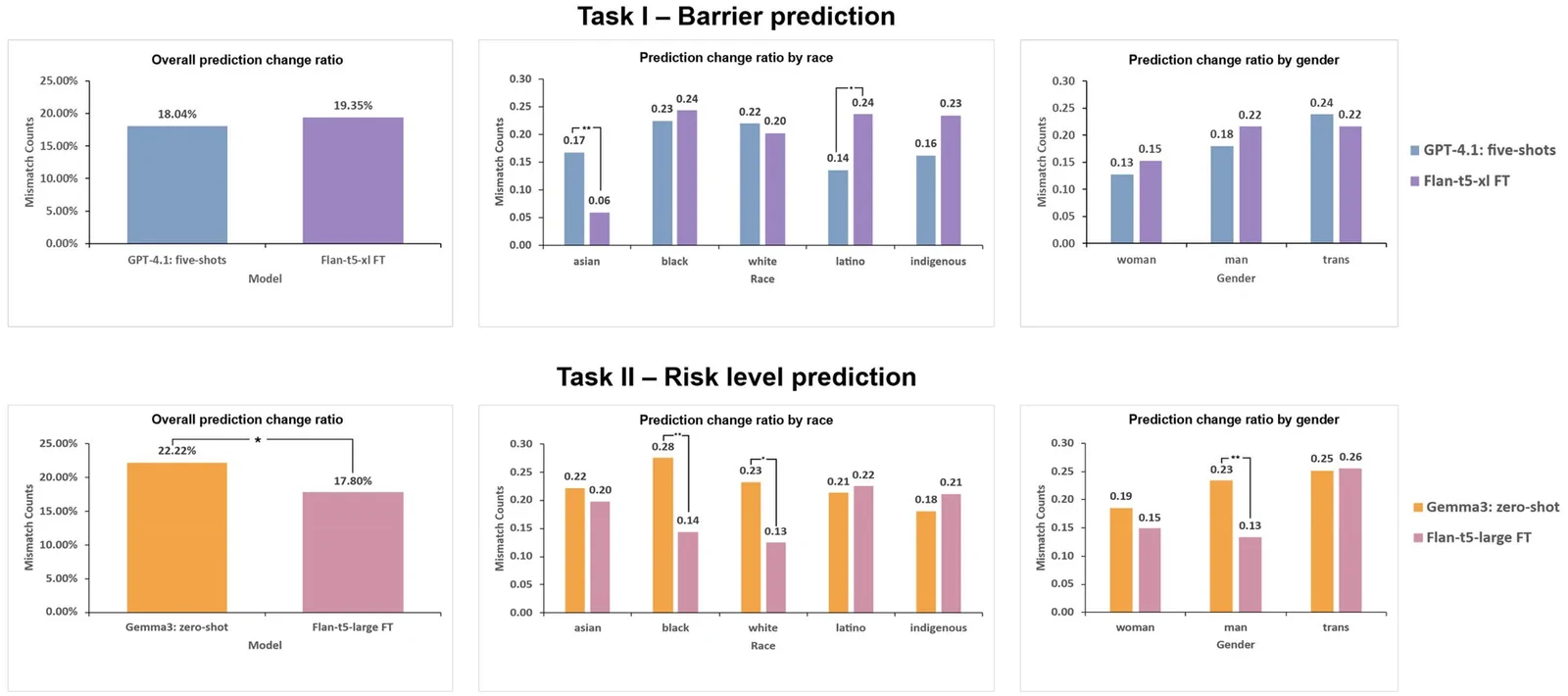
Rate of prediction changes following the injection of race and gender descriptors. Results are shown across race/ethnicity and gender for Task I—barrier detection and Task II—risk level detection. One asterisk (*) indicates statistical significance at *P < *.05, and 2 asterisks (**) indicate *P* < .01 based on chi-squared tests conducted with each subgroup.

Figure 3 shows the rate of prediction changes following the injection of race and gender descriptors.

For barrier detection, the overall mismatch rate for fine-tuned Flan-T5-xl was 19.35% (162/837), comparable to GPT-4.1 with 5-shot prompting (18.04%, 151/837). However, GPT-4.1 exhibited significantly greater prediction instability for the “Asian” descriptor compared with Flan-T5-xl (*P = .*003), while Flan-T5-xl was more sensitive to the “Latino” race (*P = .*03). No significant differences were observed for gender-related prediction changes.

**Figure 4.**
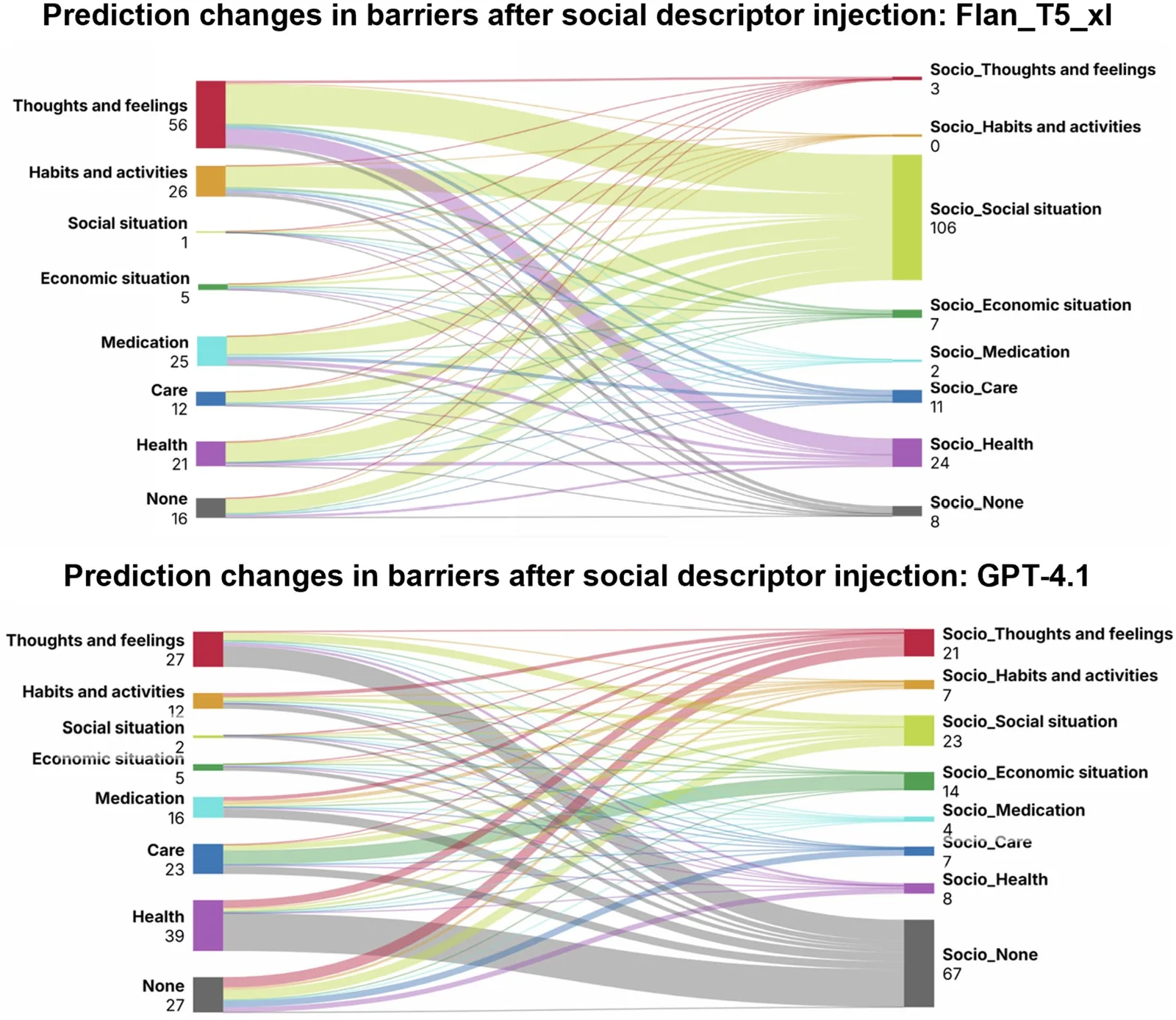
Shifts in predicted barrier types following racial and gender descriptor injection. Left: Before injection; right: after injection.

**Figure 5.**
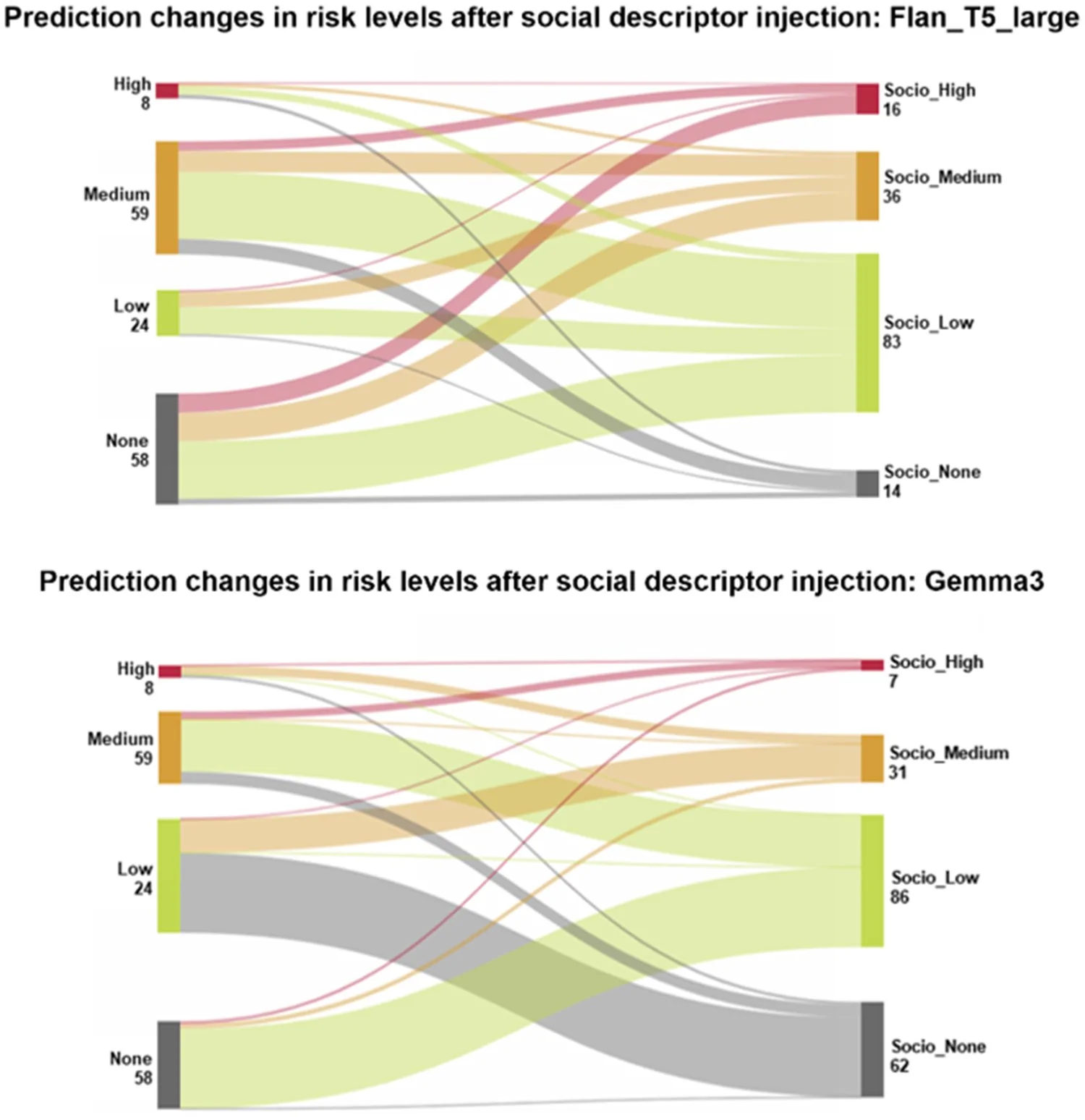
Shifts in predicted risk levels following racial and gender descriptor injection. Left: Before injection; right: after injection.

Figures 4 and 5 show the shifts in predicted barrier types and risk levels before and after sociodemographic descriptor injection.

Following descriptor injection, the majority of Flan-T5-xl mismatches shifted to the *Social Situation* category (106/162, 65%), while GPT-4.1 mismatches most frequently shifted to *None* (67/151, 44%).

For risk level detection, Flan-T5-large demonstrated a significantly lower overall mismatch rate (17.80%, 149/837) compared to Gemma3-12B with zero-shot prompting (22.22% (186/837), *P* = .024). Gemma3-12B exhibited significantly higher mismatch rates with “Black” (*P = .*006), “White” (*P = .*016), and “Man” (*P = .*003) descriptors. Most mismatches for Flan-T5-large shifted to the *Low* (83/149, 56%) or *Medium* risk levels (36/149, 24%), while Gemma3-12B mismatches most frequently shifted to the *Low* (86/186, 46%) or *None* risk categories (62/186, 33%).

Estimated energy consumption and carbon emissions during model fine-tuning and inference are detailed in Table S6 in the Supplementary File 2. As expected, energy use increased with model size. Fine-tuning Flan-T5-xl for barrier prediction consumed 1.217 kWh of energy and generated 0.573 kg CO2eq, approximately twice that of Flan-T5-large (0.604 kWh, 0.274 kg CO2eq) and over 10 times that of smaller models such as BERT-base (0.091 kWh, 0.043 kg CO2eq). A similar trend was observed for risk level detection tasks.

For inference, Flan-T5-xl consumed 0.013 kWh per 1000 inferences, double that of Flan-T5-large (0.007 kWh). In comparison, Gemma3-12B required 0.043 kWh—over 3 times more than Flan-T5-xl—and GPT-4.1 consumed an estimated 0.376 kWh,^73^ nearly 30 times that of Flan-T5-xl. Across models, carbon emissions were influenced not only by energy use but also by hosting location, with lower CO2eq reported for models hosted in the Netherlands and Canada, reflecting cleaner energy sources.

## Discussion

We developed a set of LLM-based triage models to identify ART adherence barriers and associated risks in patient messages. Larger Flan-T5 models demonstrated better generalization than smaller BERT-based models across both tasks. We also observed that fine-tuned general-domain models generally outperformed clinical foundation models of similar size and architecture, although this advantage was not consistent, and they exceeded the performance of GPT and other open-source LLMs using zero- and 5-shot CoT prompting. Finally, the fine-tuned models showed lower sensitivity to the injection of gender and racial descriptors, particularly for risk level prediction, and offered a more sustainable option for deployment, requiring less energy for inference compared to other LLMs.

Our models demonstrated robust discriminatory performance in classifying ART adherence barriers and associated risk levels from unstructured, patient-generated text, achieving Macro-F1 scores of 0.83 and 0.80 on the test split, with corresponding scores of 0.71 and 0.57 on the external validation dataset. To our knowledge, this is the first study to address medication adherence challenges directly from free text, in contrast to prior AI-based adherence models that have primarily relied on structured data such as EHRs.^22^^,^^74^^,^^75^ Most existing models also frame adherence prediction as a binary classification task aimed at predicting overall adherence status (adherent/non-adherent), with reported F1 scores ranging from 0.75 to 0.80.^22^^,^^74^^,^^75^ Despite tackling a more complex, multi-class problem, our best-performing model Flan-T5-large achieved comparable or superior performance (F1: 0.90 on the test split; 0.76 on the external validation). Beyond performance, binary classification provides a limited perspective, as individuals may be adherent while still experiencing underlying barriers that increase their risk of future non-adherence. By simultaneously identifying specific barrier types and stratifying risk levels, our LLM-based approach may provide a more nuanced understanding of individual challenges, supporting tailored, timely interventions.^76^ A scoping review of HIV guidelines from high-income countries found no consensus on adherence assessment methods or frequency, with clinical assessment every 3-6 months after ART initiation or renewal being most commonly proposed.^77^ Yet clinical visits may not reliably capture adherence barriers, whether due to time constraints or patients’ reluctance to disclose. Continuous monitoring of patient-generated messages could bridge this gap by providing a complementary, patient-initiated channel for earlier barrier detection and prompt delivery of guideline-recommended responses (education, barrier-specific support, and provider referral) between increasingly spaced clinical visits, before consequences on clinical outcomes emerge. Nonetheless, the current model classifies messages reporting perfect adherence despite explicit barriers as “None,” as no imminent nonadherence risk is inferred. From a clinical perspective, future work could therefore explore longitudinal monitoring to flag PWH who report barriers despite maintained adherence, before nonadherence occurs.

Model performance showed minimal differences between general-domain models of varying sizes on the primary test split, but generalization gaps became evident on external datasets—a common phenomenon in which smaller models are more prone to overfitting.^78^ With advances in resource-efficient adaptation methods such as LoRA, developing task-specific larger models has become more cost-effective, making them a practical option for healthcare settings where computing infrastructure is often limited.^79^ Category-level performance was highest for the *None* category across both tasks (F1 = 0.92 for Task I, 0.91 for Task II), likely reflecting its greater representation in the training data. Categories characterized by clear, concrete language, such as *Medication*, *Care*, and *Health*, also performed well (F1 = 0.90, 0.92, and 0.94, respectively). In contrast, categories requiring subjective interpretation, such as *Thoughts & Feelings* and *Social Situation*, showed greater variability (F1 = 0.73 and 0.79, respectively). For instance, a sentence reflecting a social stigma-related adherence barrier was misclassified by the model as *Habits & Activities* based on the presence of the keyword “travel.” Misclassification within *Thoughts & Feelings* was also common, as negative emotions often co-occur with other barrier types, leading to overlap and misclassification; in risk prediction, a sentence emphasizing the severe consequences of non-adherence—reflecting one’s strong motivation rather than actual risk—was incorrectly classified as *High* risk. These errors reflect the known limitations of LLMs in tasks involving implicit reasoning,^80^ often due to shortcut learning, where models rely on surface cues rather than deeper context.^81^^,^^82^ From a clinical standpoint, not all error types carry the same consequence. A key distinction lies in the direction of misclassification. Errors assigning an incorrect category or risk level still prompt clinical attention and are relatively tolerable. False negatives, however, are more problematic, as subtle risk signals or an emerging barrier to care may go undetected, potentially delaying intervention before treatment interruption or virological failure occurs. For annotation-related errors, inconsistent labels are expected to decrease with further annotation iterations, while ambiguous inputs and cross-category cases reflect inherent clinical complexity that is unlikely to be fully resolved through annotation alone. This mirrors the interpretive uncertainty clinicians themselves often face with short, context-limited patient messages.^15^^,^^17^ Among model-related errors, contextual misinterpretation may be mitigated through multi-sentence or document-level classification that better captures the patient’s broader narrative, while semantic overlap and implicit expressions may improve as LLMs continue to develop stronger reasoning capabilities.^83^ Future work should explore whether such approaches enhance the detection of these more complex, clinically nuanced signals in practice.

Another key factor limiting category-level performance was data imbalance, particularly for *Economic Situation* and *High* risk. To mitigate this challenge, we used annotation guideline-guided prompts with multiple LLMs to augment the training data. However, the results were generally low-quality and highly homogeneous, requiring re-annotation and validation. For *Economic Situation*, augmenting its training share from 1% to 3% moderately improved performance (for Flan-T5-xl: 0.67, > Care = 0.58), though it still fell short of well-represented categories. Data scarcity remains a recognized challenge in clinical AI.^84^ Our study also incorporated the integration of qualitative interview transcripts into the training data—a rich source of domain-specific data that, to the best of our knowledge, is rarely leveraged in clinical NLP tasks. While promising, further research is needed to build more efficient and scalable approaches to address data scarcity in such scenarios.

While domain-specific pretraining is expected to confer advantages for downstream tasks,^39–41^ our results challenge this assumption. In both adherence barrier and risk classification tasks, clinical foundation models performed significantly worse than general-domain models after fine-tuning on the primary test split (*P* < .001 across all model pairs) and showed no clear advantage on the external validation set (ΔMacro-F1 ranging from  + 0.01 to −0.13). A likely explanation lies in the nature of our data—conversational, informal, and user-generated—which differs substantially from the biomedical literature or EHRs typically used for clinical pretraining. Moreover, ART medication adherence is shaped not only by biomedical factors, but also by personal experiences and social contexts,^85^ which are better captured in everyday language. Similar findings have been reported by van Buchem et al. in modeling depression in cancer patients’ day-to-day communication.^35^ These results echo concerns in the clinical AI community regarding the real-world utility of clinical foundation models.^42^ Despite their theoretical promise, their practical value may be limited when tasks fall outside narrowly defined clinical knowledge domains. When developing models for tasks involving user-facing, real-world health communication, fine-tuning clinical foundation models may not be the optimal choice.

The fine-tuned general-domain models also significantly outperformed GPT-4.1 and other open-source LLMs on both tasks (min Δ Macro-F1 = 0.09 for barrier detection and 0.07 for risk level detection; *P* < .001). Although a 5-shot chain-of-thought prompting strategy guided by our annotation framework was applied, all evaluated models, including DeepSeek-R1 and Qwen3 with reasoning capabilities, performed poorly, even underperforming the BERT-base model. Interestingly, GPT-4.1 achieved the highest F1 score (0.50) for the *High* risk category, indicating some ability to detect serious adherence risks, consistent with prior findings.^45^ Nevertheless, the overall performance gap suggests the limitations of deploying off-the-shelf LLMs for this task and reinforces the need for task-specific model development.

In assessing model bias, our best-performing fine-tuned model and GPT-4.1 demonstrated comparable rates of prediction mismatches for adherence barriers following the injection of racial and gender descriptors (19% vs 18%). This responsiveness can be beneficial, as adherence barriers may manifest differently depending on the individual’s social identity. Fairness in this context should be understood as equitable reasoning across social groups, rather than equal accuracy alone. Notably, 65% of the mismatches in our model shifted to the *Social Situation* category—closely linked to HIV-related stigma—suggesting that the model may be appropriately sensitive to relevant social factors. In contrast, nearly half of GPT-4.1's mismatches were reclassified as *None*, raising concerns about false negatives that could lead to missed opportunities to identify barriers. For risk prediction, Gemma3 exhibited a 25% higher mismatch rate than our model. Predictions shifted more significantly in response to *Black*, *White,* and* Man* descriptors compared with our model, echoing concerns that LLMs may amplify biases, prejudices, and racism present in the language they are trained on.^68^ Gemma3 also reclassified approximately 30% of affected sentences to the *None* risk category, compounding the false negative concern. Taken together, these findings suggest that our fine-tuned model is more robust to demographic qualifiers than the compared LLMs. That said, descriptor injection remains an artificial stress test and may not reflect real-world language use. Future evaluations should incorporate participatory assessments with individuals from diverse racial and gender backgrounds to better assess model fairness in authentic contexts. Given the diversity of the PWH population, ensuring that LLM-based triage models are developed and deployed equitably with attention to bias is critical to avoid perpetuating health disparities. In contexts where intersecting stigma related to race, gender, and HIV status already creates barriers to care,^48^^,^^49^ bias-aware model design is not merely a technical consideration but an ethical imperative, as tools that systematically misclassify signals for racialized or gender-diverse populations risk amplifying the very disparities they are intended to address.

Finally, the environmental footprints of model development followed expected trends, with energy consumption increasing alongside model size. For barrier prediction, Flan-T5-xl achieved a modest performance gain over Flan-T5-large on the external dataset (Δ Macro-F1 = 0.03) but consumed more than twice the energy (1.217 kWh vs 0.604 kWh), equivalent to running a MacBook Air continuously for 24 hours. For risk prediction, the smaller Flan-T5-large demonstrated better generalization performance while consuming less energy, highlighting the tradeoffs between model size, performance, and sustainability. These results raise an important question: Are the marginal performance gains of larger models sufficient to offset the environmental costs? A similar tradeoff was observed during inference, where both fine-tuned models were substantially more energy-efficient than proprietary GPT-4.1, reinforcing the deployment advantages of smaller, task-specific models. Interestingly, although Gemma3 consumed more energy per inference than Flan-T5-xl, its carbon footprint was lower due to its hosting on Quebec’s low-carbon energy grid. These findings underscore the importance of considering both model selection and deployment infrastructure in clinical AI development: in high-income countries, many healthcare institutions lack the infrastructure to deploy large proprietary models locally^86^; in low- and middle-income countries, where the majority of PWH reside, these constraints are more pronounced.^87^ As clinical AI continues to scale and global HIV programs face tightening resources,^88^ the case for energy-efficient, low-cost, locally deployable, task-specific models becomes both practical and ethical.

We acknowledge several limitations of this study. First, the annotation process was labor-intensive, requiring great manual effort from patient partners, clinical experts, and researchers. Despite iterative guideline refinement, human labeling errors were inevitable. Risk annotation was particularly challenging, as it requires subjective judgment and domain-specific knowledge, compounded by the lack of consensus on adherence thresholds in clinical guidelines.^77^ Messages mentioning barriers without explicit adherence-level information required annotator interpretation, introducing potential subjectivity bias. This subjectivity likely contributed to the lower inter-rater agreement for risk labeling and may partly explain the weaker generalization of risk prediction models on external datasets. While LLMs such as GPT-4 have been proposed to assist with automated labeling,^89^^,^^90^ our experiments showed that GPT-4 achieved only moderate agreement with human annotations, underscoring the current limitations of relying on LLMs for unsupervised dataset development. Second, parts of the training data required translation from French to English, which may have introduced linguistic biases. Third, our single-label classification framework constrains the model to one category per sentence, which may not fully reflect the multidimensional nature of adherence barriers. Current models can identify the corresponding frequencies of different barriers and risks within a message. Future research will explore the frequencies and interrelationships of barriers and risks to better personalize appropriate interventions, as well as the application of multi-label classification to better reflect this complexity.

Translating these models into clinical practice will require careful integration into existing care workflows. A tiered response logic could guide implementation: high-risk signals would escalate to a human care provider, whereas medium and low-risk signals could trigger automated, barrier-targeted self-management support delivered directly to the user without provider notification. A key challenge will be managing alert fatigue, a well-documented barrier to clinical decision support adoption.^91^ This will require thoughtfully designed notification thresholds and escalation protocols to ensure that alerts remain clinically meaningful and actionable. Ultimately, successful implementation will depend on end-user acceptability and seamless workflow integration, ensuring the system strengthens care delivery without adding to provider workload.

## Conclusion

AI can help extend the reach of overstretched healthcare professionals by automating routine functions such as triage, follow-up reminders, and adherence checks.^92^ In this study, we developed LLM-based models that can effectively detect ART adherence barriers and stratify associated risks from patient-generated text, offering a novel, scalable solution for individualized HIV care. These findings show that beyond generic capabilities, tailored LLMs can operationalize a nuanced understanding of patient challenges, with the potential to inform real-time, personalized interventions. We plan to integrate these models into our MARVIN chatbot and a patient portal, with upcoming real-world validation to assess feasibility and clinical utility. Beyond technical performance, attention must be paid to ensuring these tools equitably serve diverse populations. Future research should also explore practice-based learning, enabling continuous model improvement by cycling back to real-world data. These efforts will be critical for advancing the responsible and effective application of AI in HIV care.

## Supplementary Material

ooag169_Supplementary_Data

## Data Availability

Examples of the annotated dataset and the complete synthetic dataset used in this study are available in the Dryad Digital Repository: DOI: 10.5061/dryad.w3r22817z.

## Code availability

Code and a model training and fine-tuning notebook are available at https://github.com/yma-94/ART_Adherence.
