# Supplementary material for: Large language model-based triage to identify antiretroviral therapy adherence barriers and risk levels in patient messages
Source: JAMIA Open. 2026 Sep 26;9(5):ooag169. doi: 10.1093/jamiaopen/ooag169 (PMC13615712; doi:10.1093/jamiaopen/ooag169)
Supplement: Supplementary file 1 [file ooag169_supplementary_data.zip › Supplementary_file_1.docx]

**Medication Adherence Barriers**

**&**

**Risk levels**

for People with HIV

Annotation guidelines

Written by Yuanchao Ma & The MARVIN Chatbot Triage co-construction committee

**Introduction and purpose statement**

Non-adherence to antiretroviral therapy (ART) is a widespread and multifactorial problem associated with poorer health outcomes that can increase the cost of care and the burden on health systems. However, it has been a challenge to effectively identify factors that affect adherence to medication. This project aims to empower self-management of people with HIV develop a set of deep learning-based classification algorithms that can identify, and extract data related to patients' medication adherence barriers from messages received by a chatbot and triage them according to the level of risk.

These guidelines describe specific ART adherence barriers, based on the I-Score 7-item study, we wish to capture in the messages of patients sent to/discussed with various resources, and the methodology for annotation. In addition to identifying the presence of factors that affect adherence to ART, each barrier will have certain risk levels to further enrich our datasets.

The annotation developed in this project will subsequently be used to train natural language processing systems that can “read” patient messages, identify related ART adherence barriers they may currently be suffering from, and extract them from the conversations.

10

**Annotators should carefully read these guidelines in their entirety prior to starting annotations.**

**Annotation Task**

Each message/conversation may have sentences that represent one or more of the barriers that we have defined below. Annotators are tasked with identifying the sentences that describe these ART adherence barriers concepts. If a sentence describes a barrier concept, annotators then assign a risk level of that barrier as further detailed in the specific subsections below. Assigning the label is interchangeably referred to as “annotating” or “tagging” the sentence in these guidelines.

During the annotation process, each message/conversation is pre-split into sentences, where each annotated data point will be a sentence within a document. Most annotations will not require annotators to take in any context other than what is in the boundaries of each sentence. For instances where context outside of the sentence boundaries is needed to make an annotation judgment, we ask annotators to use their best judgment, and to defer to surface level annotation without the use of textual context.

Moreover, annotators should take in the context of the patient-chatbot/physician conversation event, as well as common sense logic. Within reason, an annotator should consider the intent of the patient for a particular sentence.

We first assume in this project that all messages come from people with HIV. If it can be ascertained that it is not from a user with HIV:

- If it is one’s partner, we will still categorize it.
- If it is someone with negative diagnostic, we will classify only PrEP-related messages.

The thought process that should be considered is, "If it is not important for the patient to say what he thinks, why did he say it?" More examples of implicit barriers are detailed below, and we ask annotators to consider common sense (not just explicit textual evidence) when labeling sentences.

Finally, a sentence can describe more than one different category of medication adherence barriers, and in some cases a sentence can describe more than one barrier within a single category. **We ask annotators to please label for the moment the most relevant barrier with the highest potential risk per relevant sentence.**

Annotators should annotate strictly according to these guidelines. We acknowledge that there are many different ways to define ART adherence barriers that our guidelines may not always capture. However, the goal of this project is to develop **consistent** annotations for the specific definitions of ART adherence barriers that are detailed below. Sentences may describe a barrier and/or risk level that may seem at first glance to align with one of our barrier categories, but do not meet our specific definitions. **In these cases, please adhere only to the definitions below.** Consistency is key because consistent labels are paramount to being able to train models that can identify and extract barrier.

**ART adherence barriers: Definitions**

The definition of ART adherence barriers is some aspect of life that may make it difficult for patients to take their HIV medications as prescribed. By "difficult", we mean skipping doses, delaying doses, or being very unpleasant while taking the medication.

In terms of barriers, we followed the original I-Score definition of seven categories and added one new category to refine the prediction task. Each category was defined as follows:

**Thoughts and feelings**

This includes acceptance of having HIV, emotions (feeling sad, anxious, etc.), medication-related knowledge and beliefs or motivation to take medication.

Example intentions include:

Ask if he/she can stop taking pills

1MT_0741	I don ’ t want to take my medication anymore !

Suicide intention

4MU_2540	i am tired of my HIV condition , just wanna end my life

Always difficult taking meds - thoughts

1MT_2763	I often feel unmotivated to take my meds

Importance of taking medication daily

1MT_0747	Can i skip my complera occasionally?

**Habits and activities**

This includes daily life (one’s schedule, priorities, etc.) or substance use (alcohol, drugs, or other substances).

Example intentions include:

Missing meds long time

1MT_1534	I haven ’ t taken my stribild for more than a week , what should I do ?

4MU_0846	I didnt take my medication during many days , what should I do

Stop meds during traveling

4MU_0452	Can I take a drug holiday from medication ?

4MU_3036	should i delay my meds if i go to san fransisco

Forgetfulness

4MU_3949	I ' d like some tips on how to stop forgetting to take my medication .

1MT_3083	Often , I forget to take my atripla

Late for meds

4MU_1013	If I miss my medication one day , can I have a double dose in the next ?

4MU_1030	Is any problem if I miss take my medication just one day ?

**Social situation**

This includes personal relationships or the experience of stigma.

Example intentions include:

didn't take pill because of social stigma

3ISCORE_0604	All that I did say is that the pill I have is the flu medicine, and I decided that day I did n't take my pills, still I did n't leave because of my HIV, but it ' s just, someone who has it there .

didn't take pill because of a lot of people

3ISCORE_0595	When I travel , sometimes I ' m surrounded by several people . Sometimes , like on those days , I did n't take it .

HIV status disclosure

4MU_3683	Should I tell my sexual partner that I am HIV positive ?

4MU_4376	Do I have to tell the customs department that I have HIV ?

3ISCORE_0127	On the face of it , you ca n't tell him , well , look , I ' m , I ' m HIV positive .

Meds claiming during travel

4MU_3704	How do I hide my medication when I travel ?

relationship problem

3ISCORE_5863	Is there anything other than HIV pills , with HIV treatment that ’ s , there was a part of that decision to sort of have you say stay at home and be quiet ?

3ISCORE_1396	There is a lot of trusting, I don ’ t trust .

**Economic situation**

This includes financial or housing issues.

Example intentions include:

financial constraints

4MU_1210	One of the problem is financial constraints

3ISCORE_1579	Ah , you do n't have any food at home .

3ISCORE_1675	I ask for amounts of money every week

medication costs

4MU_0780	How much it costs medication in Canada ?

4MU_1080	It is cheap to taka hiv treatment ?

support group

4MU_1138	Montreal HIV support group

3ISCORE_1586	There are always organizations to help me

prep costs

4MU_2418	how much does prep cost

Jobless

3ISCORE_0214	Right now , I ' m off work too .

Food access-related problems are a barrier in the ***economic situation***.

**Medication**

This includes the HIV medication’s side effects, instructions or physical features (for example, pill size or taste).

Example intentions include:

Best time for taking meds?

1MT_1124	Should I take my ART before going to bed instead of the next morning ?

4MU_3873	I take my medication in the evening , but at what time is it recommended ?

change treatment

4MU_0853	I do n't like my medication , can I switch to another one ?

4MU_3871	I can take another antiretroviral treatment without being mine .

Drug interaction

4MU_1391	The medication have some interactions with protein or another supplement ?

4MU_4555	Is ginseng compatible with Juluca ?

side effects

4MU_4570	do my medications increase the risk of developing certain cancers ?

Double meds

4MU_4479	i took my medication yesterday at 20:00 , and when i woke up i did n't know if i had taken it , so i took another one , and then i remembered that i had really taken one the day before . what should i do ?

**Care**

This includes issues with healthcare professionals, the clinic, the pharmacy, or payment of medication or care.

Example intentions include:

no more treatment

4MU_0364	Can I change my medication by another medication for one time if I don ’ t have any left .

4MU_0922	I only have 3 of my 4 antiretroviral medications while travelling for two weeks .

3ISCORE_0200	There , it was particular that I forgot , that I did n't have enough pills for four days .

where to get the treatment

4MU_4017	Where can I buy my pills ?

alternatives to prep

1MT_3891	What are the other preventive measures in addition to PrEP ?

1MT_3889	What other things can I do after taking prep that will help me lower my risk of hiv infection ?

care access

4MU_3376	where can i find an HIV health provider in Montreal ?

3ISCORE_1003	Because right now , we seem to be , it ' s as if I never had a family doctor .

**Health**

This includes lab test results, HIV symptoms or overall health.

Example intentions include:

general health - emergency

4MU_0280	Am not able to get the stuff like first add kit

problematic - health

4MU_0880	I have a fever , can I take the medication ?

4MU_0829	I am feeling sick , where should I go ?

Reproductive health

4MU_0357	Can I breastfeed my baby if I ' m HIV positive ?

4MU_3308	what kind of sex can i have if i ' m HIV positive ?

How to know if undetectable

4MU_4179	If I start taking my medication , how long will it take for my viral load to become undetectable ?

U=U Type of sex

4MU_4666	sexual relations if I am undetectable

4MU_0412	Can I have oral sex with my partner if I have an undetectable viral load ?

**None**

This includes all sentences that do not mention any barrier-related information.

**Risk levels: Definition**

We defined adherence level based on the number of nonadherent days within a 30-day period:

Perfect adherence: No missed doses

Optimal adherence: ≥95% and <100% (i.e., ≤1 missed dose)

Near-optimal adherence: <95% and ≥80% (i.e., 2–5 missed doses, not occurring consecutively)

Suboptimal adherence: <80% (i.e., ≥6 missed doses)

Thus, we further define the following risk level classification scheme to assess the severity and frequency of adherence-related challenges in the message:

High: Explicit mention of an adherence barrier that leads, or may lead, to suboptimal adherence.

Medium: Explicit mention of adherence barrier that leads, or may lead, to near-optimal adherence.

Low: Explicit mention of an adherence barrier that leads, or may lead, to optimal but not perfect adherence.

None: No mention of adherence barriers, or explicit mention of a barrier with an explicit statement of perfect adherence.

See Table 1 for illustrative examples of adherence risk levels.

Table 1. Illustrative examples of adherence risk levels.

| **Example** | **Barrier** | **Risk level** |
| --- | --- | --- |
| And that might mean another 5 days of not taking my medications, cause I will not go back to the pharmacy until my next day off. | Care | High |
| So, you’re going out and sometimes it’s going to make you forget about your treatment. | Habits & Activities | Medium |
| I can't think of anything yet that could stop me, maybe if I was really sick. | Health | Low |
| I didn't see any difference, because between taking a small tablet and a bigger one at the same time, or taking just one, it doesn't make much difference. | Medication | None |
