# Supplementary material for: Large language model-based triage to identify antiretroviral therapy adherence barriers and risk levels in patient messages
Source: JAMIA Open. 2026 Sep 26;9(5):ooag169. doi: 10.1093/jamiaopen/ooag169 (PMC13615712; doi:10.1093/jamiaopen/ooag169)
Supplement: Supplementary file 1 [file ooag169_supplementary_data.zip › Supplementary_file_2.docx]

**Hyper-parameters for model training**

During the model development, the fine-tuning process of the best-performing models follows specific hyper-parameters using one A100 GPU from Google Colab.

A training batch size of 32 was used for BERT family, T5-small and -base.

For T5-large and -xl, auto_find_batch_size was set to “True” for training batch size.

Learning rate was equal to 3e-5 for BERT family, and models were trained for a total of 7 epochs.

Learning rate was equal to 1.1e-4 for T5-small and -base, and models were trained for a total of 10 epochs.

Learning rate was equal to 4e-4 for T5-large and -xl, and models were trained for a total of 10k steps.

Additionally, the LoRA configuration was employed with a rank 'r' of 16 and a 'lora_alpha' value of 32. This configuration targeted the "q" and "v" modules in the transformer layers and incorporates a dropout rate of 0.1.

lora_config = LoraConfig(

      task_type = "SEQ_CLS",

      r=16,

      lora_alpha=32,

      target_modules=["q", "v"],

      lora_dropout=0.1,

      bias= 'none',

  )

**Data augmentation – LLMs candidates and prompting strategies:**

model_list = {

"gpt_4_1": "gpt-4.1",

"llama_3_2": "llama3.2:3b",

"gemma3": "gemma3:12b",

"mistral": "mistral:latest",

"phi4": "phi4:latest",

"mixtral": "mixtral:8x7b",

}

prompt_generation = '''

Your goal is to create diverse and realistic samples for the indicated class while adhering to the task description.

**Task description**: [ We are developing a healthcare chatbot to converse with people with HIV or those at risk. The goal here is to develop a triage algorithm to identify medication adherence problems and the associated risk level. Here's the definition of each category and risk level.

We define eight categories:

1. Thoughts and feelings. This includes acceptance of having HIV, emotions (feeling sad, anxious, etc.), medication-related knowledge and beliefs, or motivation to take medication.

2. Habits and activities. This includes daily life (one’s schedule, priorities, etc.) or substance use (alcohol, drugs, or other substances).

3. Social situation. This includes personal relationships or the experience of stigma.

4. Economic situation. This includes financial or housing issues.

5. Medication. This includes the HIV medication’s side effects, instructions, or physical features (for example, pill size or taste).

6. Care. This includes issues with healthcare professionals, the clinic, the pharmacy, or payment of medication or care.

7. Health. This includes lab test results, HIV symptoms, or overall health.

8. None. This includes all sentences that do not mention any barrier-related information.

Regarding the risk level, we first define adherence level based on the number of nonadherent days within a 30-day period:

-	Perfect adherence: No missed doses

-	Optimal adherence: ≥95% and <100% (i.e., ≤1 missed dose)

-	Near-optimal adherence: <95% and ≥80% (i.e., 2–5 missed doses, not occurring consecutively)

-	Suboptimal adherence: <80% (i.e., ≥6 missed doses)

Then we define four levels: high, medium, low, and none.

1)	High: Explicit mention of an adherence barrier that leads, or may lead, to suboptimal adherence.

2)	Medium: Explicit mention of adherence barrier that leads, or may lead, to near-optimal adherence.

3)	Low: Explicit mention of an adherence barrier that leads, or may lead, to optimal but not perfect adherence.

4)	None: No mention of adherence barriers, or explicit mention of a barrier with an explicit statement of perfect adherence.

**Instructions**:

- Analyze the task description and identify key attributes or features for each class.

- Use reasoning to ensure the synthetic data aligns with the characteristics of the task and its real-world relevance.

- Generate 50 synthetic samples for **[Specific barrier]**, in JSON format, following this:

Format the output as a JSON array, not a list, and output nothing more. The objects have the keys:

'data', 'barrier', 'risklvl'. \

The 'data' should be a realistic text sample that represents the class. Ensure the sample is diverse in terms of text length and content.

The 'barrier' should be one of the nine categories defined above.

The 'risklvl' should be one of the four levels defined above.

- Ensure the samples are diverse, balanced, and representative of each class. Also, ensure that the examples are sufficiently life-like and that the wording is sufficiently colloquial, with enough details.

Give me only a nested JSON object, like {'synthetic_data': [{...}]}, where ... are the keys defined above.

'''

**Classification – LLMs prompting strategies:**

input = [

\#	{"role": "system", "content": "You are an annotator, please provide labels for the given sentences considering the following definition."},

\#	{"role": "user", "content": prompt + text}

\#	],

**For zero-shot:**

prompt = prompt_classification_zero_shot

prompt_classification_zero_shot = '''

Regarding adherence barrier, we define eight categories:

1. Thoughts and feelings. This includes acceptance of having HIV, emotions (feeling sad, anxious, etc.), medication-related knowledge and beliefs, or motivation to take medication.

2. Habits and activities. This includes daily life (one’s schedule, priorities, etc.) or substance use (alcohol, drugs, or other substances).

3. Social situation. This includes personal relationships or the experience of stigma.

4. Economic situation. This includes financial or housing issues.

5. Medication. This includes the HIV medication’s side effects, instructions, or physical features (for example, pill size or taste).

6. Care. This includes issues with healthcare professionals, the clinic, the pharmacy, or payment of medication or care.

7. Health. This includes lab test results, HIV symptoms, or overall health.

8. None. This includes all sentences that do not mention any barrier-related information.

Regarding the risk level, we first define adherence level based on the number of nonadherent days within a 30-day period:

-	Perfect adherence: No missed doses

-	Optimal adherence: ≥95% and <100% (i.e., ≤1 missed dose)

-	Near-optimal adherence: <95% and ≥80% (i.e., 2–5 missed doses, not occurring consecutively)

-	Suboptimal adherence: <80% (i.e., ≥6 missed doses)

Then we define four levels: high, medium, low, and none.

1)	High: Explicit mention of an adherence barrier that leads, or may lead, to suboptimal adherence.

2)	Medium: Explicit mention of adherence barrier that leads, or may lead, to near-optimal adherence.

3)	Low: Explicit mention of an adherence barrier that leads, or may lead, to optimal but not perfect adherence.

4)	None: No mention of adherence barriers, or explicit mention of a barrier with an explicit statement of perfect adherence.

**Instructions**:

- Analyze classification definition descriptions to identify key attributes or features of each category.

- Use reasoning to ensure that the predicted labels match the categorical definitions.

- Predict the corresponding barrier category first and then predict the risk level for the input, in JSON format, following this:

Format the output as a JSON array, not a list, and output nothing more. The objects have the keys:

"barrier", "risklvl".

The "barrier" should be your prediction from one of the eight categories defined above.

The "risklvl" should be your prediction from one of the four levels defined above.

Don't give me ANY explanation. Give me ONLY a nested JSON object, like {"prediction": [{...}]}, where ... are the keys defined above.

Now let's think step by step, generate the predictions, and output the results in JSON format.

Here is the input: {input}

'''

**For five-shots:**

prompt = prompt_classification_five_shot

prompt_classification_five_shot = '''

Regarding adherence barrier, we define eight categories:

1. Thoughts and feelings. This includes acceptance of having HIV, emotions (feeling sad, anxious, etc.), medication-related knowledge and beliefs, or motivation to take medication.

2. Habits and activities. This includes daily life (one’s schedule, priorities, etc.) or substance use (alcohol, drugs, or other substances).

3. Social situation. This includes personal relationships or the experience of stigma.

4. Economic situation. This includes financial or housing issues.

5. Medication. This includes the HIV medication’s side effects, instructions, or physical features (for example, pill size or taste).

6. Care. This includes issues with healthcare professionals, the clinic, the pharmacy, or payment of medication or care.

7. Health. This includes lab test results, HIV symptoms, or overall health.

8. None. This includes all sentences that do not mention any barrier-related information.

Regarding the risk level, we first define adherence level based on the number of nonadherent days within a 30-day period:

-	Perfect adherence: No missed doses

-	Optimal adherence: ≥95% and <100% (i.e., ≤1 missed dose)

-	Near-optimal adherence: <95% and ≥80% (i.e., 2–5 missed doses, not occurring consecutively)

-	Suboptimal adherence: <80% (i.e., ≥6 missed doses)

Then we define four levels: high, medium, low, and none.

1)	High: Explicit mention of an adherence barrier that leads, or may lead, to suboptimal adherence.

2)	Medium: Explicit mention of adherence barrier that leads, or may lead, to near-optimal adherence.

3)	Low: Explicit mention of an adherence barrier that leads, or may lead, to optimal but not perfect adherence.

4)	None: No mention of adherence barriers, or explicit mention of a barrier with an explicit statement of perfect adherence.

**Instructions**:

- Analyze classification definition descriptions to identify key attributes or features of each category.

- Use reasoning to ensure that the predicted label match the categorical definition.

- Predict the corresponding barrier category first and then predict the risk level for the input, in JSON format, following this:

Format the output as a JSON array, not a list, and output nothing more. The objects have the keys:

"barrier", "risklvl".

The "barrier" should be your prediction from one of the eight categories defined above.

The "risklvl" should be your prediction from one of the four levels defined above.

Just give me the closest set of barriers and risk levels. If there are two sets that apply, give me the one with the highest risk level. Don't give me your explanation. Give me ONLY a nested JSON object, like {"prediction": [{...}]}, where ... are the keys defined above.

**Examples**:

Here are several classification examples:

1. "I've been drinking all week, so I haven't taken my medication all week either. Am I screwed?"

- The output should be:

{"prediction": [{"barrier": "Habits and activities", "risklvl": "High"}]}

2. "I sometimes feel unmotivated to take my meds, can i skip my complera occasionally? "

- The output should be:

{"prediction": [{"barrier": "Thoughts and feelings", "risklvl": "Medium"}]}

3. "At the very beginning , the constraint was that we had to see the doctor quite often , because of research protocols."

- The output should be:

{"prediction": [{"barrier": "Care", "risklvl": "Low"}]}

4. "I'm undetectable now, so everything is fine for me."

- The output should be:

{"prediction": [{"barrier": "Health", "risklvl": "None"}]}

5. "To get the medication , you do n't necessarily have the money."

- The output should be:

{"prediction": [{"barrier": "Economic situation", "risklvl": "High"}]}

Now let's think step by step, generate the predictions, and output the results in JSON format.

Here is the input: {input}

'''

**Demographic descriptor injection – LLMs prompting strategies:**

prompt_socio_adding = """

Randomly add one of race/ethnicity [asian, black, white, latino, indigenous] and gender [woman, man, trans] description into the given sentences as natural as you can, and put the modified demographic description in bracket at the beginning, like these examples:

1. If I'm HIV positive , what is the risk of contamination ? =>

    As a [latino male] latino man, if I'm HIV positive, what is the risk of contamination ?

2. Is this a symptom of hiv ? =>

    Is this a symptom of hiv ? I am an indigenous trans [indigenous tran].

3. I ' m very afraid of hiv. =>

    I ' m a white man [white male] and I'm very afraid of hiv.

4. Can you enlighten me I ' m very afraid . =>

    Can you enlighten me, as a black woman [black female] I ' m very afraid

Now, please think step by step, and generate me just the new sentences with the indicators:

"""

Table S1. Illustrative examples of nonadherence risk levels.

| **Example** | **Barrier** | **Risk level** |
| --- | --- | --- |
| And that might mean another 5 days of not taking my medications, cause I will not go back to the pharmacy until my next day off. | Care | High |
| So, you’re going out and sometimes it’s going to make you forget about your treatment. | Habits & Activities | Medium |
| I can't think of anything yet that could stop me, maybe if I was really sick. | Health | Low |
| I didn't see any difference, because between taking a small tablet and a bigger one at the same time, or taking just one, it doesn't make much difference. | Medication | None |

Table S2. Best-performing fine-tuned model performance: binary analysis.

| **Task I - Barrier prediction** |  |  |  |  |
| --- | --- | --- | --- | --- |
|  | Dataset | Mean F1 (95% CI) | Barrier (n=840) | *None* (n=540) |
| Flan-T5-xl | Primary test split | 0.94 (0.93-0.95) | 0.95 | 0.92 |
|  | External validation | 0.85 (0.82-0.88) | 0.96 | 0.74 |
| **Task II - Risk level prediction** |  |  |  |  |
|  | Dataset | Mean F1 (95% CI) | Risk (n=615) | *None* (n=765) |
| Flan-T5-large | Primary test split | 0.90 (0.88-0.92) | 0.89 | 0.91 |
|  | External validation | 0.76 (0.72-0.79) | 0.86 | 0.65 |

*Note.* The 95% CIs for F1 scores were calculated using 300 bootstrap samples with replacement on three distinct datasets, ensuring a SE of the CI limits below 0.01. The observed SE interval limit was 0.0033. CI: confidence interval; SE: standard error.

Table S3. Analysis of primary error types.

| Types | | Examples | Reference label:  (Barrier, Risk level) | Model prediction:  (Barrier, Risk level) |
| --- | --- | --- | --- | --- |
| **Annotation-related errors** | | | | |
|  | Ambiguous or vague inputs | Medication, it’s everyday? | Medication,  Medium | Thoughts & Feelings, None |
|  |  | I want information about atripla. | Thoughts & Feelings, Low | Medication,  Low |
|  | Cross-category cases | Because the hospital, the clinic, these places are not confidential. | Social Situation,  Low | Care,  Medium |
|  |  | When I travel, sometimes I'm surrounded by several people. Sometimes, like on those days, I didn't take it. | Social Situation,  High | Habits & Activities, High |
|  | Inconsistent annotation | sometimes it's a problem. | None,  None | Thoughts & Feelings, Medium |
| **Model-related errors** | | | | |
|  | Semantic overlap | Let's say it's a dinner with friends, I could always put a little reminder in my phone, like a little alarm telling me not to forget to take my medication, even if you're having dinner with lots of people, because I can go out and take my medication, and that's that. | Social Situation,  Low | Habits & Activities, None |
|  |  | Are drug holidays safe? | Thoughts & Feelings, High | Habits & Activities, Medium |
|  |  | It's the morning ones I forget, but that has nothing to do with HIV treatment, it's the other condition. | None,  None | Habits & Activities, Medium |
|  | Implicit expression | Because if I don't take the medication, I can even die. | Thoughts & Feelings, None | Thoughts & Feelings, High |
|  |  | So, just in case it might happen that, so someone says: “What pill is that?”, I say: “it ' s a headache pill .” | Social Situation,  Low | Medication,  Low |
|  | Contextual misinterpretation | Artificial dye has done hard on the kidneys. | Medication,  Medium | Health,  Low |
|  |  | That is a huge question to me. | Thoughts & Feelings, High | None,  None |

Table S4. Best-performing fine-tuned model vs GPT & other open-source LLMs - performance on external validation dataset (Section A)

| **Task I - Barrier prediction** | | | | | | | | | | | | |
| --- | --- | --- | --- | --- | --- | --- | --- | --- | --- | --- | --- | --- |
| Model | Parameters (Total/Tuned) | Mean Macro F1 (95% CI) | ΔMacro-F1 | *P* value | Thoughts & Feelings (F1) | Habits & Activities (F1) | Social Situation (F1) | Economic Situation (F1) | Medication (F1) | Care (F1) | Health (F1) | None (F1) |
| **Flan-T5-xl** | 3B/9.4M | **0.71 (0.68-0.74)** |  |  | **0.69** | **0.76** | **0.66** | 0.67 | **0.74** | 0.58 | **0.83** | **0.74** |
| **Five-shot** | Total parameters | | | | | | | | | | | |
| **GPT-4.1** | N/A | **0.62 (0.59-0.65)** | -0.09 | <.001 | 0.54 | 0.70 | 0.63 | 0.66 | 0.73 | 0.64 | 0.57 | 0.51 |
| GPT-4.1-mini | N/A | 0.59 (0.56-0.63) | -0.12 | <.001 | 0.52 | 0.60 | 0.61 | 0.76 | 0.65 | 0.64 | 0.51 | 0.47 |
| GPT-4.1-nano | N/A | 0.60 (0.57-0.63) | -0.11 | <.001 | 0.45 | 0.66 | 0.53 | 0.75 | 0.66 | **0.71** | 0.58 | 0.49 |
| Gemma3 | 12B | 0.59 (0.56-0.63) | -0.12 | <.001 | 0.51 | 0.42 | 0.57 | 0.74 | 0.65 | 0.58 | 0.74 | 0.55 |
| Qwen3 | 8B | 0.60 (0.57-0.63) | -0.11 | <.001 | 0.52 | 0.62 | 0.63 | **0.83** | 0.67 | 0.60 | 0.50 | 0.43 |
| Mistral | 7B | 0.58 (0.54-0.61) | -0.13 | <.001 | 0.52 | 0.54 | 0.52 | 0.74 | 0.55 | 0.59 | 0.65 | 0.49 |
| DeepSeek-R1 | 7B | 0.45 (0.42-0.49) | -0.26 | <.001 | 0.39 | 0.46 | 0.26 | 0.70 | 0.46 | 0.37 | 0.52 | 0.49 |
| LLaMA3.2 | 3B | 0.51 (0.48-0.55) | -0.20 | <.001 | 0.48 | 0.47 | 0.37 | 0.66 | 0.51 | 0.43 | 0.67 | 0.53 |
| **Zero-shot** |  |  |  |  |  |  |  |  |  |  |  |  |
| GPT-4.1 | N/A | 0.60 (0.56-0.63) | -0.11 | <.001 | 0.45 | 0.70 | 0.63 | 0.66 | 0.73 | 0.60 | 0.51 | 0.50 |
| GPT-4.1-mini | N/A | 0.56 (0.53-0.60) | -0.15 | <.001 | 0.52 | 0.65 | 0.59 | 0.75 | 0.67 | 0.56 | 0.38 | 0.43 |
| GPT-4.1-nano | N/A | 0.57 (0.54-0.60) | -0.14 | <.001 | 0.44 | 0.66 | 0.48 | 0.69 | 0.64 | 0.66 | 0.52 | 0.47 |
| Gemma3 | 12B | 0.60 (0.56-0.63) | -0.11 | <.001 | 0.52 | 0.58 | 0.57 | 0.72 | 0.66 | 0.57 | 0.66 | 0.50 |
| Qwen3 | 8B | 0.55 (0.51-0.58) | -0.16 | <.001 | 0.44 | 0.64 | 0.53 | 0.82 | 0.67 | 0.54 | 0.33 | 0.41 |
| Mistral | 7B | 0.50 (0.46-0.53) | -0.21 | <.001 | 0.47 | 0.44 | 0.38 | 0.72 | 0.46 | 0.49 | 0.62 | 0.40 |
| DeepSeek-R1 | 7B | 0.39 (0.35-0.42) | -0.32 | <.001 | 0.44 | 0.48 | 0.22 | 0.69 | 0.36 | 0.18 | 0.34 | 0.40 |
| LLaMA3.2 | 3B | 0.33 (0.29-0.36) | -0.38 | <.001 | 0.35 | 0.31 | 0.32 | 0.61 | 0.31 | 0.06 | 0.25 | 0.39 |
| **Task II - Risk level prediction** | | | | | | | | | | | | |
| Model | Parameters (Total/Tuned) | Mean Macro F1 (95% CI) | Δ F1 | P value | High (F1) | Medium (F1) | Low (F1) | None (F1) |  |  |  |  |
| **Flan-T5-large** | 760M/4.7M | **0.57 (0.53-0.60)** |  |  | 0.42 | **0.65** | **0.55** | 0.65 |  |  |  |  |
| **Five-shot** | Total parameters | | | | | | | | | | | |
| GPT-4.1 | N/A | 0.43 (0.40-0.47) | -0.14 | <.001 | **0.50** | 0.13 | 0.42 | **0.67** |  |  |  |  |
| GPT-4.1-mini | N/A | 0.42 (0.39-0.45) | -0.15 | <.001 | 0.47 | 0.21 | 0.37 | 0.63 |  |  |  |  |
| GPT-4.1-nano | N/A | 0.43 (0.40-0.47) | -0.14 | <.001 | 0.47 | 0.40 | 0.23 | 0.62 |  |  |  |  |
| Gemma3 | 12B | 0.47 (0.44-0.51) | -0.10 | <.001 | 0.48 | 0.34 | 0.44 | 0.64 |  |  |  |  |
| Qwen3 | 8B | 0.33 (0.30-0.37) | -0.24 | <.001 | 0.42 | 0.13 | 0.21 | 0.57 |  |  |  |  |
| Mistral | 7B | 0.38 (0.35-0.41) | -0.19 | <.001 | 0.38 | 0.33 | 0.21 | 0.60 |  |  |  |  |
| DeepSeek-R1 | 7B | 0.35 (0.31-0.38) | -0.22 | <.001 | 0.29 | 0.27 | 0.28 | 0.55 |  |  |  |  |
| LLaMA3.2 | 3B | 0.28 (0.26-0.31) | -0.29 | <.001 | 0.24 | 0.07 | 0.24 | 0.59 |  |  |  |  |
| **Zero-shot** |  |  |  |  |  |  |  |  |  |  |  |  |
| GPT-4.1 | N/A | 0.45 (0.42-0.48) | -0.12 | <.001 | 0.46 | 0.32 | 0.39 | 0.62 |  |  |  |  |
| GPT-4.1-mini | N/A | 0.41 (0.37-0.44) | -0.16 | <.001 | 0.46 | 0.24 | 0.32 | 0.61 |  |  |  |  |
| GPT-4.1-nano | N/A | 0.43 (0.39-0.46) | -0.14 | <.001 | 0.40 | 0.44 | 0.26 | 0.61 |  |  |  |  |
| **Gemma3** | 12B | **0.50 (0.46-0.54)** | -0.07 | <.001 | 0.39 | 0.58 | 0.42 | 0.62 |  |  |  |  |
| Qwen3 | 8B | 0.30 (0.27-0.34) | -0.27 | <.001 | 0.34 | 0.11 | 0.19 | 0.57 |  |  |  |  |
| Mistral | 7B | 0.32 (0.29-0.34) | -0.25 | <.001 | 0.33 | 0.34 | 0.06 | 0.53 |  |  |  |  |
| DeepSeek-R1 | 7B | 0.30 (0.27-0.34) | -0.27 | <.001 | 0.23 | 0.19 | 0.26 | 0.54 |  |  |  |  |
| LLaMA3.2 | 3B | 0.31 (0.29-0.34) | -0.26 | <.001 | 0.21 | 0.12 | 0.39 | 0.52 |  |  |  |  |

Table 4. Best-performing fine-tuned model vs GPT & other open-source LLMs - performance on external validation dataset (Section B)

*Note.* Total parameters of the GPT4 family models were not publicly available. The 95% CIs for Macro-F1 scores were calculated using 300 bootstrap samples with replacement on three distinct datasets, ensuring a SE of the CI limits below 0.01. The observed SE interval limits was 0.0039. ΔMacro-F1 represents the Macro-F1 difference between best-performing fine-tuned general-domain models and prompt-tuned GPT and other open-source LLMs. Bold values indicate the best performance for each task among other LLMs. *P* values were calculated using the Mann-Whitney U test, with Bonferroni correction applied for multiple comparisons (adjusted significance threshold: P < 0.00625, based on adjusted α = α/m, where α = 0.05, m = 8 (number of tests, 8 models)). CI: confidence interval; SE: standard error; N/A: Not applicable.

Table S5. Most common discrepancies between ground-truth and best-performing model prediction for each task (Count>=10)

| **Task** | **Ground truth** | **Model prediction** | **Count** |
| --- | --- | --- | --- |
| **Barrier prediction** | None | Thoughts & Feelings | 20 |
|  | None | Habits & Activities | 15 |
|  | Thoughts & Feelings | Habits & Activities | 11 |
|  | Thoughts & Feelings | None | 10 |
| **Risk level prediction** | None | Low | 38 |
|  | None | Medium | 37 |
|  | Medium | None | 29 |
|  | Medium | Low | 27 |
|  | Low | None | 22 |
|  | Low | Medium | 21 |
|  | Medium | High | 12 |
|  | High | Medium | 11 |

Table S6. Estimated energy consumption and carbon footprints during model fine-tuning and inference.

|  | Model | Parameters (Total/tuned) | Energy consumed (kWh) | Carbon footprint (CO2eq, kg) | Hosted country |
| --- | --- | --- | --- | --- | --- |
| **Fine-tuning** | | | | | |
| Barrier | BERT-base | 110M/110M | 0.091 | 0.043 | Singapore |
|  | BioBERT | 110M/110M | 0.096 | 0.045 | Singapore |
|  | BioClinicalBERT | 110M/110M | 0.096 | 0.045 | Singapore |
|  | Flan-T5-small | 77M/77M | 0.074 | 0.033 | United States |
|  | Flan-T5-base | 248M/248M | 0.165 | 0.044 | Netherlands |
|  | Clinical-T5-base | 248M/248M | 0.153 | 0.041 | Netherlands |
|  | Flan-T5-large | 760M/4.7M | 0.604 | 0.274 | United States |
|  | Clinical-T5-large | 760M/4.7M | 0.532 | 0.251 | Singapore |
|  | **Flan-T5-xl** | **3B/9.4M** | **1.217** | **0.573** | **Singapore** |
| Risk Level | BERT-base | 110M/110M | 0.096 | 0.045 | Singapore |
|  | BioBERT | 110M/110M | 0.096 | 0.045 | Singapore |
|  | BioClinicalBERT | 110M/110M | 0.091 | 0.043 | Singapore |
|  | Flan-T5-small | 77M/110M | 0.074 | 0.033 | United States |
|  | Flan-T5-base | 248M/248M | 0.165 | 0.044 | Netherlands |
|  | Clinical-T5-base | 248M/248M | 0.152 | 0.041 | Netherlands |
|  | **Flan-T5-large** | **760M/4.7M** | **0.575** | **0.260** | **United States** |
|  | Clinical-T5-large | 760M/4.7M | 0.440 | 0.207 | Singapore |
|  | Flan-T5-xl | 3B/9.4M | 1.028 | 0.484 | Singapore |
| **Inference** |  |  | Energy consumed per 1,000 queries (kWh) |  |  |
| Barrier | **Flan-T5-xl** | **3B** | **0.013** | **6.00e-03** | **United States** |
|  | GPT-4.1 | N/A | 0.376 | ~0.3 | United States* |
| Risk Level | **Flan-T5-large** | **760M** | **0.007** | **3.30e-03** | **United States** |
|  | Gemma3 | 12B | 0.043 | 1.00e-04 | Canada |

*Note.* * Energy consumption and carbon emissions for GPT-4.1 were estimated based on You^1^ and Jegham et al.^2^, assuming 1,000 input tokens and 20 output tokens per query, resulting in approximately 0.376 kWh per 1,000 inferences. Carbon emissions per 1,000 tokens were estimated at ~0.3 g CO2eq based on Soham^3^. All model training was conducted using Google Colab, leading to variability in the hosting country. Inference for Gemma 3-12B was performed locally at the institution in Montreal, Quebec, Canada. Bold values indicate the best-performing fine-tuned model for each task.

1.	You J. How much energy does ChatGPT use? Feb 07, 2025 (<https://epoch.ai/gradient-updates/how-much-energy-does-chatgpt-use?utm_source=chatgpt.com>).

2.	Jegham N, Abdelatti M, Elmoubarki L, Hendawi A. How Hungry is AI? Benchmarking Energy, Water, and Carbon Footprint of LLM Inference. arXiv preprint arXiv:250509598 2025.

3.	Soham. The Cost of Inference: Running the Models. (<https://tinyml.substack.com/p/the-cost-of-inference-running-the>).
